# Transcriptional Profiling of the Dose Response: A More Powerful Approach for Characterizing Drug Activities

**DOI:** 10.1371/journal.pcbi.1000512

**Published:** 2009-09-18

**Authors:** Rui-Ru Ji, Heshani de Silva, Yisheng Jin, Robert E. Bruccoleri, Jian Cao, Aiqing He, Wenjun Huang, Paul S. Kayne, Isaac M. Neuhaus, Karl-Heinz Ott, Becky Penhallow, Mark I. Cockett, Michael G. Neubauer, Nathan O. Siemers, Petra Ross-Macdonald

**Affiliations:** 1 Bristol-Myers Squibb Research and Development, Princeton, New Jersey, United States of America; 2 Johnson Graduate School of Management, Cornell University, Ithaca, New York, United States of America; 3 Congenomics, Glastonbury, Connecticut, United States of America; Lilly Singapore Centre for Drug Discovery, Singapore

## Abstract

The dose response curve is the gold standard for measuring the effect of a drug treatment, but is rarely used in genomic scale transcriptional profiling due to perceived obstacles of cost and analysis. One barrier to examining transcriptional dose responses is that existing methods for microarray data analysis can identify patterns, but provide no quantitative pharmacological information. We developed analytical methods that identify transcripts responsive to dose, calculate classical pharmacological parameters such as the EC50, and enable an in-depth analysis of coordinated dose-dependent treatment effects. The approach was applied to a transcriptional profiling study that evaluated four kinase inhibitors (imatinib, nilotinib, dasatinib and PD0325901) across a six-logarithm dose range, using 12 arrays per compound. The transcript responses proved a powerful means to characterize and compare the compounds: the distribution of EC50 values for the transcriptome was linked to specific targets, dose-dependent effects on cellular processes were identified using automated pathway analysis, and a connection was seen between EC50s in standard cellular assays and transcriptional EC50s. Our approach greatly enriches the information that can be obtained from standard transcriptional profiling technology. Moreover, these methods are automated, robust to non-optimized assays, and could be applied to other sources of quantitative data.

## Author Summary

Transcriptional profiling is arguably the most powerful hypothesis-free method for investigating biological effects of drugs—so why do the experiments typically use outmoded single-dose designs? Such single-dose experiments will co-mingle effects that can occur with different potency (e.g., effects on the known target versus effects on additional undesired targets). Single-dose experiments have little comparability to the dose-response bioassays, which are now used throughout the drug discovery processes. One reason for the disparity between experimental approaches is that existing analytical methods for dose-response bioassays can't cope with the dimensionality of microarray data: a typical bioassay is optimized for one response, then used to run a screen against thousands of compounds; whereas transcriptional profiling measures thousands of non-optimized responses to a single compound. Conversely, existing methods for microarray data analysis can identify patterns, but provide no quantitative dose-response information. To overcome these problems, we developed novel algorithms and visualization methods that allow anyone to apply transcriptional profiling as a conventional dose-response assay. The approach provides far more information than limited-dose designs, yet is economical (12 arrays/compound). With this new analytical framework, it is now possible to identify distinct transcriptional responses at distinct regions of the dose range, to link these impacts to biological pathways, and to make realistic connections to drug targets and to other bioassays.

## Introduction

The necessity of dose information in interpreting drug effects has been recognized since the 16^th^ century, when Paracelsus observed: “All things are poison, and nothing is without poison: the dose alone makes a thing not poison” [1]. Today, dose-response models are routinely used to evaluate drug effects in biochemical and cell-based assays. Pharmacological parameters such as the widely used EC50 value (half-maximal Effective Concentration) are central to any discussion of drug activities. In contrast, transcription profiling experiments are typically performed using replicate treatments at one dose, and effects are identified by analysis of variance [2]. Single-dose experiments cannot distinguish effects that have different potencies, and they limit the utility of expression data relative to other bioassays. This is regrettable given the many applications of transcriptional profiling in drug discovery [3]–[8].

There is no inherent reason for transcription profiling not to use the dose-response designs seen in every other area of chemical biology [9]. Transcript levels are known to exhibit dose-responsive behavior in response to ligands, toxins and pharmacological agents [10]–[12]. Compound:target interaction at a single site that follows the law of mass action is reflected by the sigmoidal dose response seen in many bioassays [13]. Although the algorithms used to quantify such dose responses in optimized bioassays are not ideal for microarray data, they have been used successfully to identify dose-responsive transcripts in two studies [11],[14],[15].

While transcriptional responses are typically controlled through second messengers, it can be shown mathematically [16] and empirically [12] that when intermediate steps have the same characteristics, the sigmoidal response is preserved. An important corollary of these properties is that if a compound binds with distinct potencies to multiple targets, multiple biological responses will occur, with EC50 values corresponding to the target-binding EC50. Transcriptional profiling provides an informative genome-wide view of biological responses [17], thus obtaining quantitative dose-response information for transcript responses has obvious application in characterizing compounds that have high potential to interact with multiple targets. For example, establishing selectivity of kinase inhibitors across the human kinome continues to be difficult [18].

We describe analysis of transcription profiling studies of the dose responses to four kinase inhibitors: imatinib, nilotinib, dasatinib and PD0325901. Imatinib is a relatively selective [18] clinical ABL inhibitor; nilotinib is a similar but more potent second-generation compound [19]. Dasatinib is a highly potent clinical ABL inhibitor that has additional activities on Src family [20] and receptor tyrosine kinases [21]. PD0325901 is a non-ATP competitive inhibitor of MEK, a threonine/tyrosine kinase [22]. Like most pharmaceutical agents, these compounds bind a single site on their target and elicit sigmoidal dose responses in biochemical [23],[24] and cellular [25]–[27] assays. We developed novel methods to efficiently identify the transcripts that exhibit a sigmoidal dose response, and to visualize and further characterize groups of coordinated transcriptional responses. These analyses allow comparisons of potency, establish connection to other cellular assays, and provide insight into the mechanism and selectivity of compounds.

## Results

### Identification of dose-responsive transcripts

Experimental data were obtained from the human lung cell line A549, treated for four hours with imatinib, nilotinib, dasatinib and PD0325901. This timeframe allows both down- and up-regulated transcripts to be identified, since there is detectable mRNA turnover for the majority of transcripts in four hours [28]. For the ABL inhibitors, a 20 hour treatment was also performed. Treatments used 12 concentrations from 170 pM to 30 µM (a three-fold dilution series covering a six logarithmic range). This dose regimen is modeled on other dose-response bioassays, and is amenable to identifying responses with EC50s in the interval between 0.54 nM and 10 µM. Each of the 22,215 probesets on an Affymetrix HG-133A array was treated as an assay for the response of its corresponding transcript; the 12 intensity values for each treatment constituted the assay data. Only sigmoidal dose responses were evident in a hierarchical cluster analysis of the data (Text S1).

**Figure 1:**
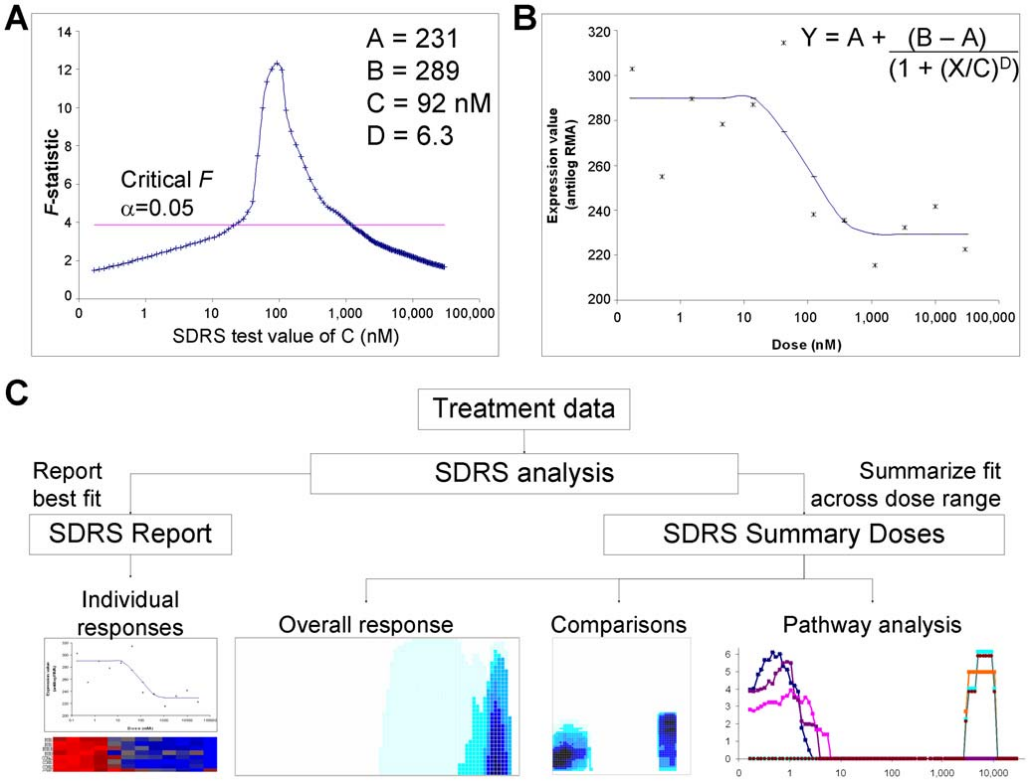
Implementation and output of SDRS algorithm. (A) SDRS analysis of intensity data for probeset 205016_at (Transforming Growth Factor Alpha), following a four hour treatment with dasatinib. The plot shows the maximal *F*-statistic obtained for this probeset at each C value evaluated by SDRS. The critical value of the *F*-distribution at *P*<0.05 is indicated. The global maximal *F* was obtained from fit of experimental data to a sigmoidal dose response model with the values of A, B, C, D shown. (B) The experimental data for probeset 205016_at, and the curve obtained using the optimal model parameters established by SDRS in the equation shown. (C) Schematic diagram of the data analysis flow. The SDRS report (left) contains the calculated pharmacological parameters for each probeset, and can be used to identify individual response transcript of interest. This report is qualitatively similar to the output of an iterative algorithm. The SDRS Summary Doses output (right) is unique to the grid search method. This dataset is used for the further approaches shown, which characterize and compare the overall transcriptional responses. A more detailed flowchart of SDRS is provided in Text S1.

A typical sigmoidal dose response curve is defined by an equation with four unknowns corresponding to minimal response (A), maximal response (B), EC50 (C), and slope (D) [13]. To identify dose-responsive transcripts, we developed a grid search-based algorithm named Sigmoidal Dose Response Search (SDRS). For each probeset, the SDRS algorithm tests a series of candidate EC50 values (C) across the dose range. The goodness of fit at every grid search dose is measured by an *F*-statistic, calculated as the ratio between mean square of regression and mean square of error. When the data for a given probeset fits a sigmoidal dose response, its *F*-statistic plot has an inverted ‘V’ shape (Figure 1A), and the values of A, B, C and D that generate the maximal *F* are the best fitting model (Figure 1B), and C approximates the true EC50 for the assay data. Given the normality of residuals (Text S1 and Table S9), the statistic follows an *F*-distribution, and *F*-tables can be used to establish significance. A probeset is designated as a ‘response transcript’ if its maximal *F*-statistic is larger than the critical value for *P*<0.05 (see Methods) In this work, this criterion for a response transcript is used for benchmarking to other algorithms, and to retrieve gene sets for study after their significance has been established with methods that employ multiple test corrections.

The performance of SDRS compared favorably to XLfit (Text S1 and Table S1), software that implements the Levenberg-Marquardt algorithm [29]. Using SDRS, response transcripts were identified for the four kinase inhibitors in the seven treatments described (Table S2).

As diagrammed in Figure 1C, one output of SDRS is qualitatively similar to that of an iterative algorithm: each probeset has a predicted EC50, *P* value and fold-change (Table S2). However, SDRS also generates an *F*-statistic for every probeset at each grid search dose level. This output, which is unique to the grid search method, allowed us to characterize and compare the coordinated transcriptional dose responses using the novel approaches described below.

### Characterization of the transcriptional dose response

SDRS identifies the subset of transcripts that exhibit sigmoidal dose response behavior, and estimates the potency of the effect. While each probeset is an independent assay, transcript levels respond to the treatment's effect on a limited number of biological targets. At doses where the treatment impacts a target, one might identify coordinated sets of transcription responses. Identifying more than one coordinated set of responses that occur at distinct doses within a treatment series would point to multi-target pharmacology of a drug. However, if one has only a single EC50 value per probeset, it is impossible to identify such coordinated responses without imposing binning criteria, which are inherently arbitrary and not amenable to statistical evaluation.

**Figure 2:**
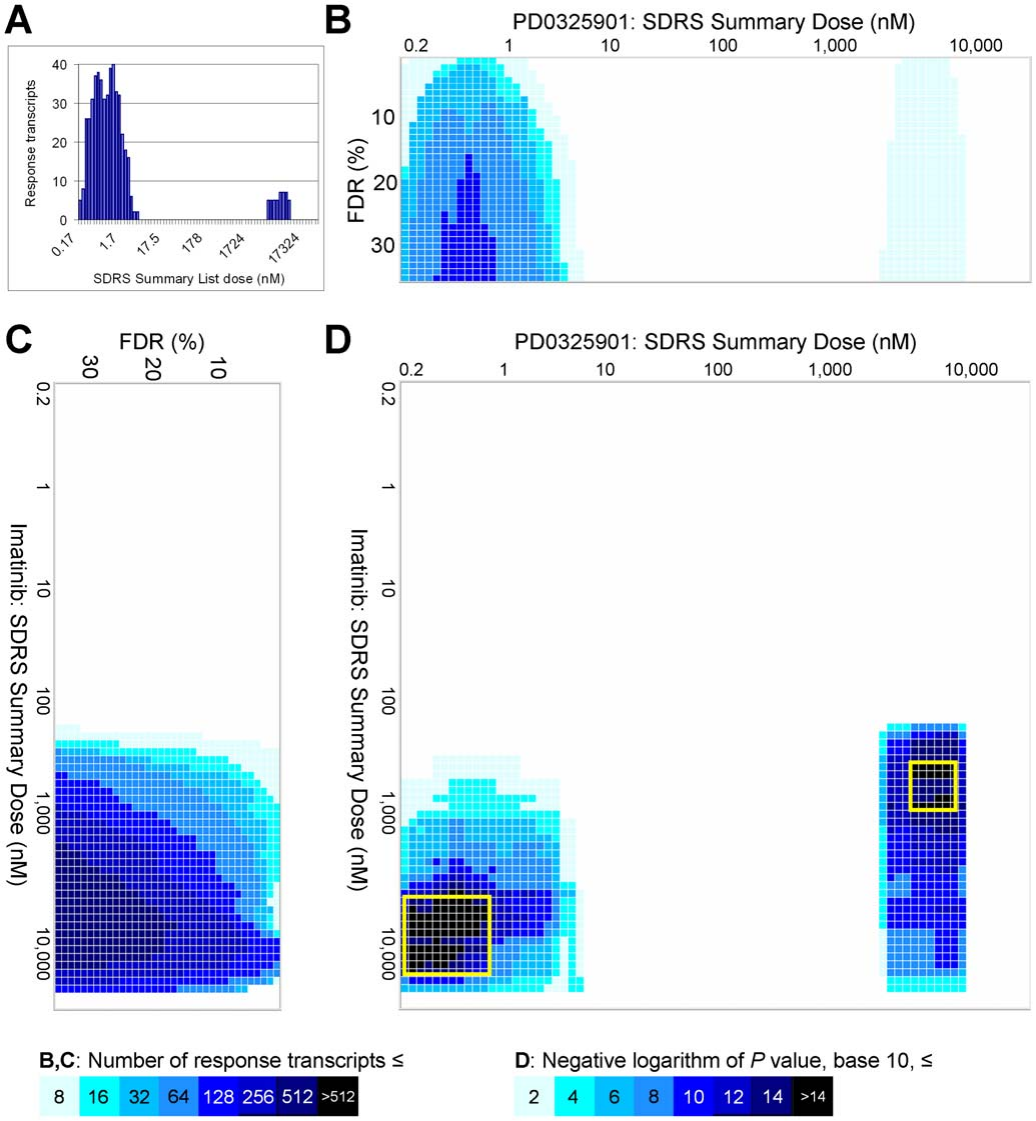
Characterization of the overall transcriptional responses to PD0325901 and imatinib, and a comparison between the two treatments. (A) Bar chart displaying the number of probesets whose *F*-statistic for goodness of fit to a sigmoidal curve passes a 10% FDR criterion at each SDRS Summary Dose. The results shown are from a four hour treatment with PD0325901. These results are also presented as the tenth row of the heatmap in (B). (B–C) Heatmaps showing the number of probesets whose *F*-statistic for goodness of fit to a sigmoidal curve passes the given FDR criterion at each SDRS Summary Dose. In (B), the horizontal axis contains the SDRS Summary Doses, and the vertical axis contains one row for each of the 35 FDR criteria applied. Panel (C) is rotated 90° to clarify its relationship to panel (D). The results shown are from a four hour treatment with PD0325901 (B) or a 20 hour treatment with imatinib (C). (D) The negative logarithm of *P* values from Fisher's exact tests that compare response transcripts for PD0325901 and imatinib. Values displayed are the lowest *P* value observed in tests performed between the (maximally) 35 lists of probesets that underlie each dose in panels (B) and (C). The horizontal and vertical axes contain the SDRS Summary Doses for PD0325901 (four hours) and imatinib (20 hours), respectively. The two boxed regions indicate shared transcript responses discussed in the text.

The SDRS output allows an alternative, statistically rigorous means to identify coordinated transcriptional responses. We exploited the fact that SDRS provides a list of the *F*-statistic for every probeset at each grid search dose. (To simplify presentation, we use only the *F*-statistic lists from ‘Summary Doses’: a log-evenly distributed subset of the SDRS search doses). A false discovery rate (FDR) correction was applied to each Summary Dose list; this multiple test correction effectively removes spurious response transcripts (Text S1 and Table S8). A bar chart of the results for a given FDR revealed ‘peaks’ at regions of the dose range, indicative of coordinated transcriptional responses (Figure 2A). Since a single FDR criterion also represents an arbitrary limitation of analysis, we computed the numbers of response transcripts corresponding to FDRs from 1% to 35%.

We devised a novel and effective visualization for this data: a heat map where columns correspond to the Summary Doses, each row is a histogram of results at a given FDR, and intensity reflects the number of response transcripts passing the FDR criterion. Thus, the data presented in Figure 2A is the tenth row of the heat map in Figure 2B. As with a histogram, ‘peaks’ were evident at distinct regions of the dose range (Figure 2B, 2C and Text S1). These peaks reflect the likelihood that a coordinated transcriptional response is occurring around a given dose. For example, the MEK-specific inhibitor PD0325901 induced two separate peaks of transcriptional responses at four hours (Figure 2B). The first peak centered at 0.5 nM and contained 80 response transcripts at 35% FDR. A second peak centered at 6 µM contained seven response transcripts. Examination of loci with multiple response transcripts suggested that the width of these peaks reflects the precision with which an EC50 for a single target can be determined (Table S3). Of the three ABL inhibitors, dasatinib induced the most potent and populous transcriptional response at both time points, consistent with its multi-kinase activity [21],[23].

### Comparison of treatments using their transcriptional dose responses

Drug treatments are typically compared by evaluating the overlap between the two lists of regulated transcripts. The SDRS output allows a similar but more granular evaluation, since one can see whether transcripts are regulated with the same potency in each treatment. At each Summary Dose there is a list of probesets that may be sorted based on *F*-score and truncated based on FDR criteria. To compare the data from two treatments, we used a Fisher's exact test on each possible pair of Summary Doses, incrementing the response transcript lists from 1% to 35% FDR (see Methods). The resulting grid of *P* values is effectively visualized as a heat map. Comparison of a treatment to itself produced low *P* values at points on the diagonal (Text S1). If two distinct treatments generate the same response transcripts but with different EC50s, low *P* values occur at points off the diagonal. For example, 4 hour treatment with PD0325901 gave a peak of transcriptional responses centered around 6 µM (Figure 2B). Imatinib affected an overlapping set of response transcripts with EC50s around 500 nM. Conversely, a set of transcripts with EC50s around 1 nM for PD0325901 had EC50s over 1 µM for imatinib (Figure 2C,D and Text S1).

### Connecting transcriptional dose response to effects on pathways and processes

The heat maps in Figure 2B,C and Text S1 provide an overview of transcriptional responses to a compound. To understand the underlying mechanism, the responding genes need to be mapped to cellular pathways. This typically involves evaluating the overlap between the regulated genes and lists of genes representing biological processes [30]. Results can be prioritized by *P*-value, usefulness of the process grouping and relevance to possible targets. The SDRS output allows a similar but more granular evaluation of pathway impact, since one can see the dose at which the cellular process is implicated. For each treatment, the FDR-corrected response transcript list at each Summary Dose was tested for overlap with lists of genes connected to processes in the KEGG [31] and Gene Ontology (GO, [32]) databases (see Methods and Table S4). For processes meeting a *P*<0.001 criterion, impact on a discrete pathway at a distinct region of the dose range was used to prioritize the following examples.

**Figure 3:**
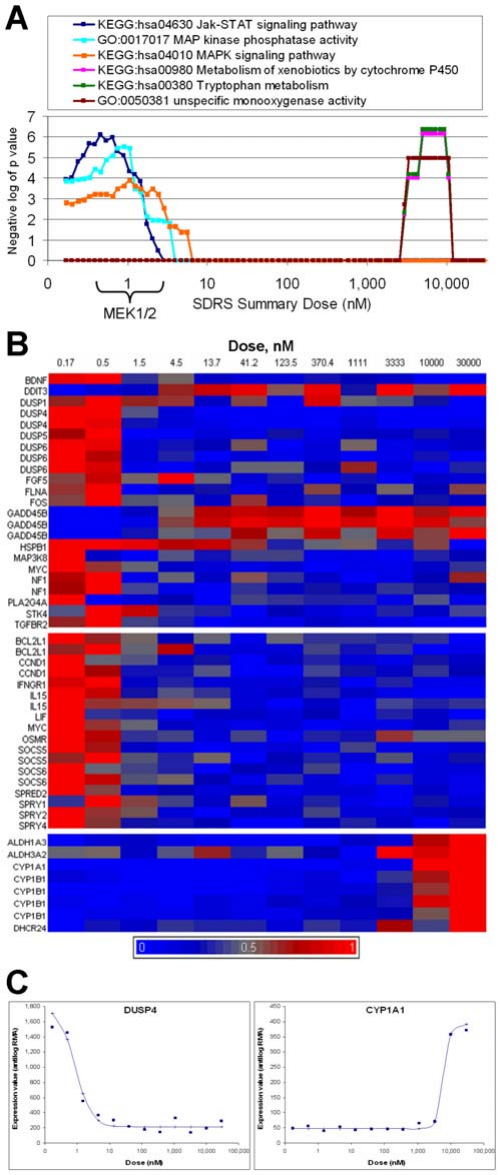
Pathway analysis applied to the transcriptional response to PD0325901 at four hours. (A) Plot of significance values obtained from Fisher's exact tests performed between the lists of gene loci corresponding to response transcripts (1 to 35% FDR), and lists of gene loci representing the six biological pathways indicated. PD0325901 causes ∼50% inhibition of cellular MEK activity in the dose region indicated [33]. (B) Signal intensity for 48 response transcripts (in rows) identified by SDRS, across twelve doses of PD0325901 (columns) at 4 hours. Top panel contains transcripts related to MAPK signaling (KEGG:hsa04010). Middle panel contains transcripts related to Jak-STAT signaling (KEGG:hsa04630). Bottom panel contains transcripts mapped to the pathways for metabolism of xenobiotics by cytochrome P450 (KEGG:hsa00980) and tryptophan metabolism (KEGG:hsa00380). For visualization, RMA signal intensity data was reverse-logged and scaled from zero (blue) to one (red) for each probeset. (C) Experimental data for probesets corresponding to DUSP4 (204014_at, EC50 0.9 nM, left panel), annotated to the pathway for MAPK signaling (KEGG:hsa04010), and CYP1A1 (205749_at, EC50 6.0 µM, right panel), annotated to the pathway for metabolism of xenobiotics by cytochrome P450 (KEGG:hsa00980), with dose response curves obtained from the optimal model parameters established by SDRS.

For PD0325901, plotting the results of this analysis (Table S4, Figure 3A) indicated that the two peaks of transcriptional responses observed in Figure 2B represented distinct biological effects. PD0325901 specifically inhibits cellular MEK activity with an IC50 of around 1 nM in both Ras/Raf-mutant and wild-type cell lines [33]. The response transcripts with EC50s around this dose were enriched for components of the MAP kinase pathway, which contains MEK (Figure 3B, upper panel, Table S5) and the Jak-STAT pathway (Figure 3B, middle panel), which is reported to be indirectly affected by MEK function [34]–[36]. These response transcripts included MAP kinase phosphatases (DUSP1, DUSP4, DUSP5, and DUSP6) [37] and STAT regulators (IFNGR1, OSMR) and targets (BCL2L1, CCND1, MYC, LIF, IL15, SOCS5, SOCS6) [38]–[40] (Figure 3B,C). In contrast, the response transcripts with EC50s around 6 µM comprised 5 genes (CYP1A1, CYP1B1, ALDH1A3, ALDH3A2, and DHCR24; Figure 3B lower panel, Figure 3C), all mapped to the P450-mediated xenobiotic metabolism and tryptophan metabolism pathways. This transcriptional response was also provoked by treatment with imatinib at around 500 nM (Figure 2D, and Table S4), and could reflect a xenobiotic response to these compounds, or another shared target activity.

**Figure 4:**
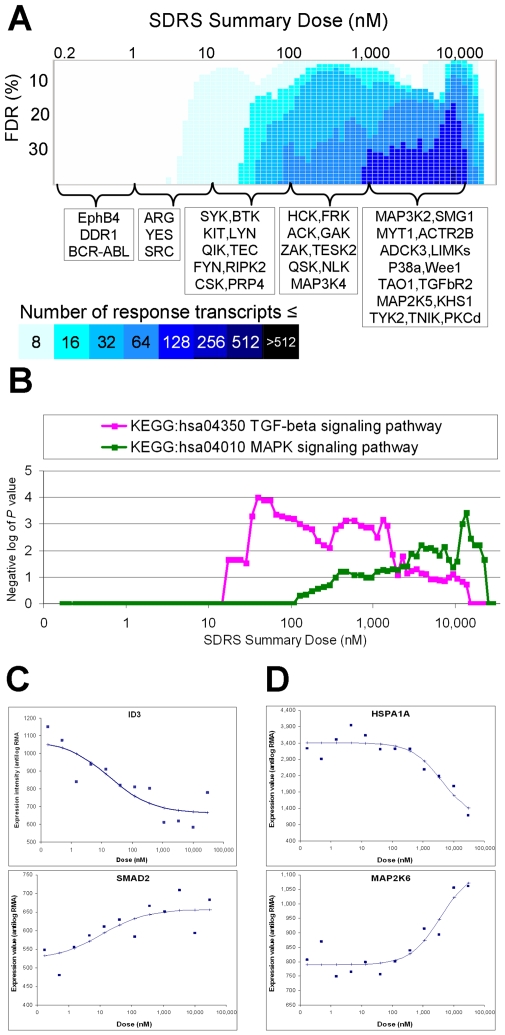
Pathway analysis applied to the transcriptional response to dasatinib at four hours. (A) Heatmap showing the number of probesets whose *F*-statistic for goodness of fit to a sigmoidal curve passes the given FDR criterion at each SDRS Summary Dose. Known kinase targets of dasatinib are shown below, with the dose interval for their binding EC50 in cells [41]. (B) Significance values obtained from Fisher's exact tests performed between the lists of gene loci corresponding to response transcripts (1 to 35% FDR), and lists of gene loci representing the three biological pathways indicated. (C) Experimental data for probesets corresponding to ID3 (207826_s_at; EC50 17.5 nM) and SMAD2 (203077_s_at; EC50 10.7 nM) annotated to the TGF-beta signaling pathway (KEGG:hsa04350), with the dose response curves obtained from the optimal model parameters established by SDRS. (D) Experimental data for probesets corresponding to HSP1A1 (200799_at; EC50 3.9 µM) and MAP2K6 (205698_s_at; EC50 3.6 µM) annotated to the MAPK signaling pathway (KEGG:hsa04010), with the dose response curves obtained from the optimal model parameters established by SDRS.

Dasatinib had a complex transcriptional response across the dose range (Figure 4A), reflecting its potent impact on numerous kinases [21]. Combining dose information with pathway analysis allows scientists to make realistic connections between these target kinases and responses. For example, at Summary Doses from 34–128 nM there is an enrichment of transcripts for genes in the TGF-β signaling pathway, including the ID repressor family and SMADs (Figure 4B,C). However since dasatinib's cellular activity on TGF-β family kinases is in the micromolar range ([41], shown in Figure 4A) they are not the relevant target. Instead, hypotheses should include the kinases that dasatinib does inhibit in this dose region: some are known to impact the TGF-β pathway, for example Src [42]. Another example is the impact on MAP kinase signaling at higher concentrations of dasatinib (Figure 4B,D). Dasatinib's impact was distinct from that of PD0325901 by both automated comparison (Text S1), and by direct analysis of the response transcript lists (Table S5). PD0325901 predominantly affected loci assigned to the ‘classical’ MAP kinase pathway containing its target, MEK. Dasatinib regulated transcripts for a different set of loci in the ‘classical’ pathway, where it targets EGFR and Raf kinases. Dasatinib also regulated transcripts for loci in the KEGG p38/Jnk pathway, where it targets p38α, MLTK (ZAK), and TGFβR2. The potency of the transcript responses was consistent with known potencies for these targets ([21],[41], see Figure 4A and Table S5).

**Figure 5:**
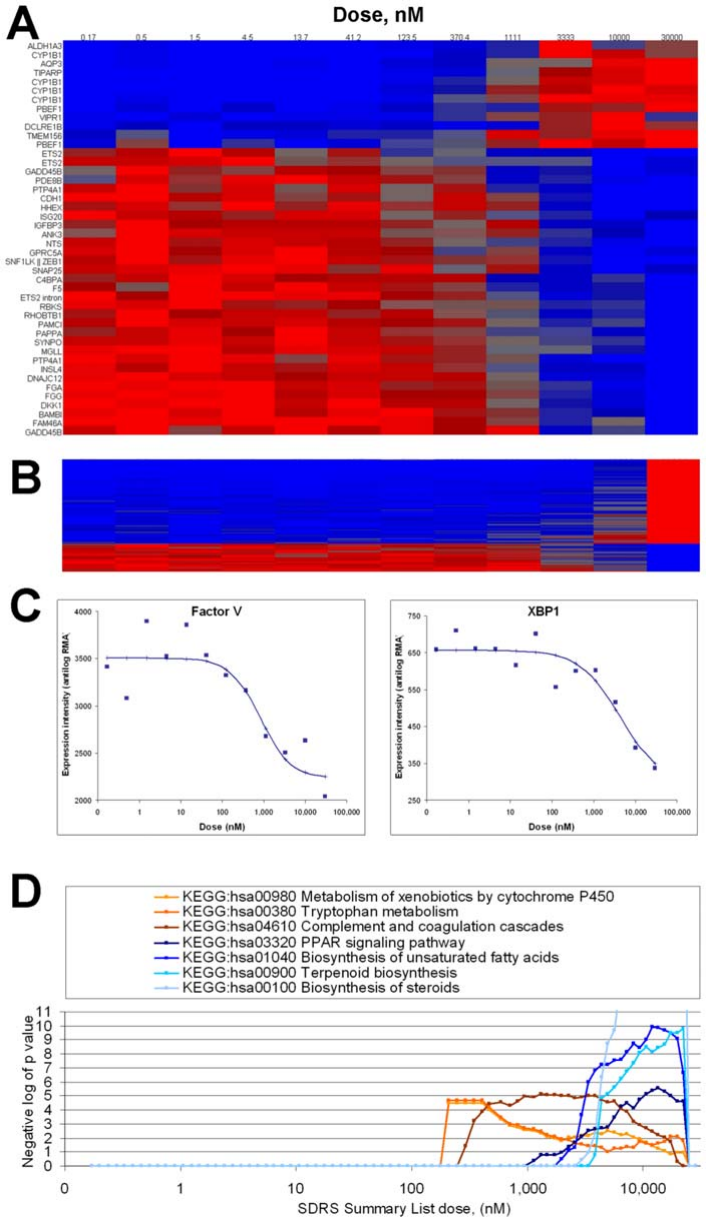
Dose-dependence and pathway analysis of the transcriptional response to imatinib at 20 hours. (A, B) Signal intensity for 245 SDRS response transcripts (FDR10%, >1.5-fold change; in rows) across twelve doses of imatinib (columns) following 20 hours of treatment. (A) contains the subset of 44 response transcripts with EC50<3 µM. (B) contains the subset of 201 response transcripts with EC50>3 µM. For visualization, RMA signal intensity data was reverse-logged and scaled from zero (blue) to one (red) for each probeset. (C) Experimental data for probesets corresponding to Factor V (204713_s_at; EC50 824 nM) and XBP1 (200670_at, EC50 4.2 µM), with the dose response curves obtained from the optimal model parameters established by SDRS. (D) Significance values obtained from Fisher's exact tests performed between the lists of gene loci corresponding to response transcripts (1 to 35% FDR), and lists of gene loci representing the seven KEGG biological pathways indicated.

For imatinib, dose-dependent effects have been invoked to explain discrepancies between pre-clinical studies [43]. In examining imatinib's transcriptional responses at 20 hours, we used the efficacious clinical plasma concentration of 3 µM [44] as a reference point. Of 245 response transcripts for imatinib (10% FDR, >1.5-fold change, Table S2), only 44 have EC50s<3 µM (Figure 5A, C). The remaining 201 response transcripts have EC50s>3 µM (Figure 5B). The 201 response transcripts include 35 for endoplasmic reticulum-localized proteins such as XBP1 (Figure 5C), supporting observations that *in vitro* treatments with imatinib at 5 µM affect the function of this compartment [45]. Pathway analysis identified enrichment of transcripts for the seven KEGG pathways (*P*<0.0001) shown in Figure 5D. Transcripts with EC50s>3 µM had enrichment for several pathways affecting lipid metabolism, indicating distinct biological impacts as the dose increases beyond the required clinical range.

Submicromolar doses of imatinib, dasatinib and nilotinib did not produce shared effects on transcription (Text S1), indicating that their shared potent inhibition of cellular targets such as ABL and PDGFR [23],[41] does not provoke transcriptional responses in the A549 cell line. In the micromolar dose range, comparison of transcriptional responses indicated shared effects at 20 hours (Text S1). Pathway analysis indicated both shared and compound-specific effects in this dose range (Text S1).

### Connecting transcriptional dose responses to other dose response assays

**Figure 6:**
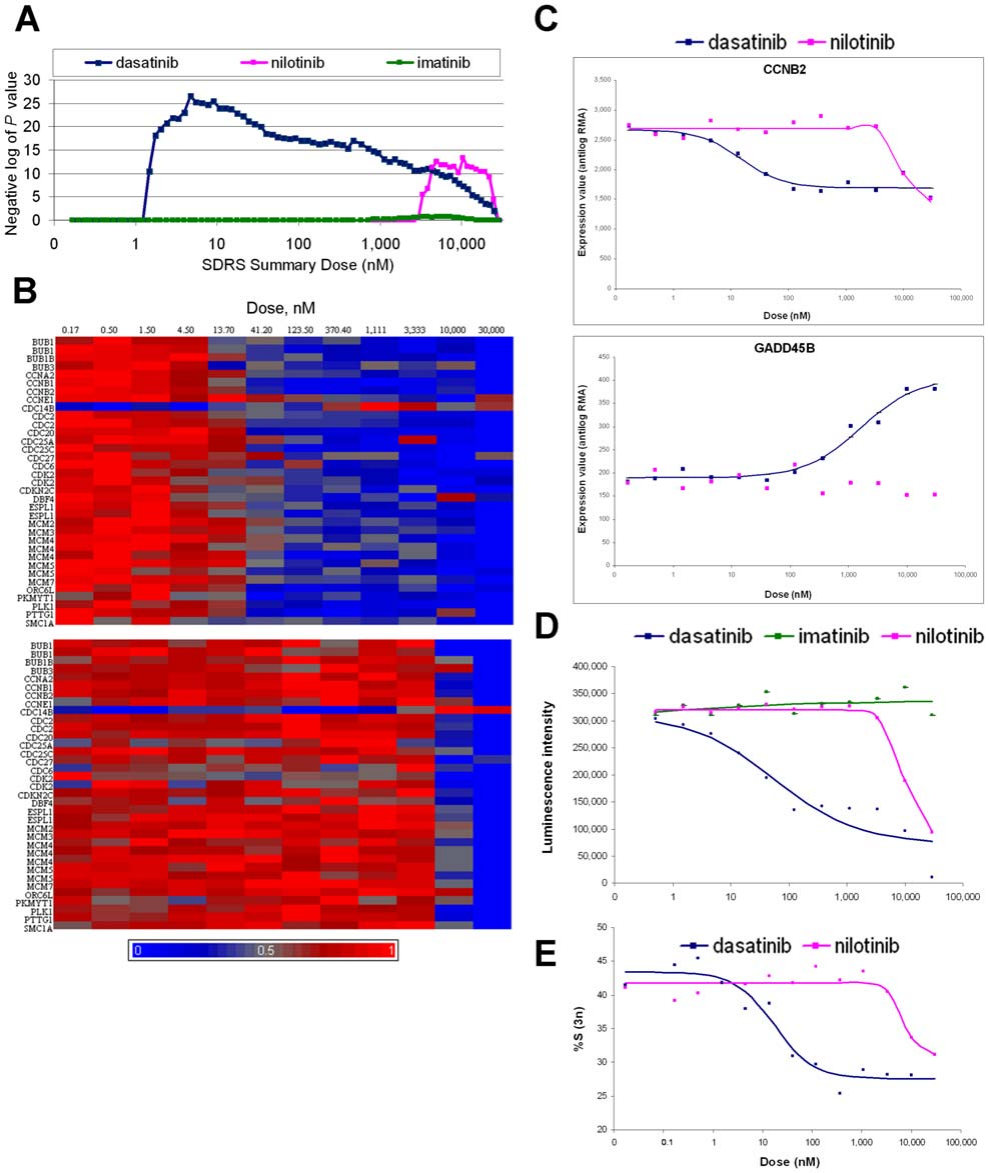
Dose-dependence of impact on cell cycle for dasatinib, nilotinib and imatinib. (A) Significance values obtained from Fisher's exact tests performed between the lists of gene loci corresponding to response transcripts at each SDRS Summary Dose, and lists of gene loci implicated in cell cycle regulation (KEGG:hsa04110). (B) Probeset signal intensity data from a 20 hour treatment with dasatinib (upper panel) or nilotinib (lower panel) at the doses indicated. Rows contain data for 35 response transcripts that correspond to genes implicated in cell cycle regulation (KEGG:hsa04110). For visualization, RMA signal intensity data was reverse-logged and scaled from zero (blue) to one (red) for each probeset. (C) Signal intensity data for CCNB2 (202705_at; EC50 14.8 nM for dasatinib and 9.3 µM for nilotinib) and GADD45B (207574_s_at; EC50 1.7 µM for dasatinib, no curve fit for nilotinib), upon treatment with dasatinib or nilotinib for 20 hours at the doses indicated. The dose-response curves obtained from the optimal model parameters established by SDRS are shown. (D) Luminescence assay for ATP content performed on cell cultures treated with dasatinib, nilotinib or imatinib for 96 hours at the doses indicated. (E) Percentage of the treated cell population in S phase (3n DNA content) in cell cultures treated with dasatinib or nilotinib for 23 hours at the doses indicated. The full data set is in Table S7.

Pathway analysis found that a significant number of genes involved in the cell cycle (KEGG pathway hsa04110) were represented in the transcriptional responses to dasatinib and nilotinib but not imatinib at 20 hours (Figure 6A and Table S4). The broad peak for dasatinib reflects impact at two distinct dose regions, based on the distribution of EC50s for individual response transcripts (Table S6). The first group of 44 response transcripts has EC50s around 10 nM and includes the DNA helicase complex, cyclins and CDKs (Figure 6B,C). The second group of 35 response transcripts has EC50s in the micromolar range and includes known transcriptional targets of p53: GADD45 (Figure 6C), CDKN1A and Stratifin [46], PCNA and MDM2. Pathway analysis also confirmed dasatinib's impact on DNA replication (KEGG:hsa03030) and p53 signaling (KEGG:hsa04115); Table S4). By contrast, nilotinib impacts the cell cycle pathway in one dose region: 62/63 of the response transcripts have EC50s in the micromolar range (Table S6). While nilotinib also regulates transcripts for the DNA helicase complex, cyclins and CDKs, it does not have a significant impact on the p53 signaling pathway (Figure 6B,C).

To compare these findings from transcriptional profiling with typical cell-based assays for proliferation, we treated our A549 cell line with the 12-point dose range of dasatinib, imatinib, and nilotinib and assayed the ATP content of samples (a surrogate for cell number) at 96 hours, and the DNA content of dasatinib and nilotinib samples (a surrogate for cell-cycle stage) at 23 hours. The results reflected our findings from pathway analysis: dasatinib and nilotinib inhibited proliferation and decreased the S phase population, whereas imatinib did not (Figure 6D,E). Importantly, the potency in these conventional measures of cell cycle impact agreed with the EC50s of transcripts for the DNA helicase complex, cyclins and CDKs (EC50s around 10 nM for dasatinib and 6 to 9 µM for nilotinib).

## Discussion

This paper is the first description of a systematic application of genome-wide transcriptional profiling as a traditional dose-response assay. Since most compounds act on multiple targets with different potencies, target-specific effects of a compound may not be distinguished by the limited dose selection of a typical transcriptional profiling experiment. We show that with a dose-response study design that uses just 12 arrays per compound, one can use the existing technology in a more informative way, and establish connection to other cellular dose-response assays.

We present new algorithms and visualization methods that allow one to identify, compare, and characterize transcriptional responses. Sigmoidal dose responses are usually identified by iterative nonlinear regression methods [29]. SDRS applies nonlinear regression in a grid search, and performs equally well. It is robust against natural variability, and will be amenable to identifying dose responses in other sources of quantitative data. The most important benefit of SDRS over iterative regression or clustering methods is obtaining the fitting statistic (*F*) across the dose range for each transcript. This provides a moving window to evaluate the transcriptome's coordinated responses across the dose range. The full set of *F*-statistics permitted the further methods we present, which easily characterize and compare the overall transcriptional dose response with statistical rigor.

Our results demonstrate that transcription profiling has many of the properties of traditional dose-responsive bioassays that have been used for decades [47]. The ability to combine dose information from diverse bioassays with dose-dependent pathway analysis proves valuable in connecting transcriptional responses with targets. For example, the MEK-specific inhibitor PD0325901 produced a significant transcriptional response at the known cellular potency for MEK inhibition [33], and had no further effect on transcription until micromolar doses. In contrast, the transcriptional response to dasatinib had multiple EC50s, consistent with its known activities on multiple targets [21]. Nonetheless pathway analysis allowed us to map cellular processes to distinct regions of the dose range, and connect them to likely kinase targets identified in other dose-response cellular assays [41]. Connection to discrete kinase inhibition events can be refined by numerous methods: kinases not expressed in the experimental cell line can be excluded from lists of hits from biochemical assays, and comparisons can be made with transcript response profiles for compounds that have overlapping target spectra, or with profiles generated following siRNA ablation of kinase targets.

The power of a dose response study design stems from the ability to rigorously compare pharmacological parameters across assays [48]. For example, we show that PD0325901 affects STAT-regulated transcripts with an EC50 of 1 nM, the same potency as its cellular activity on MEK [33], and this connectivity provides compelling support for earlier reports that the ERKs are STAT kinases [34]. We also show connectivity between the EC50s for transcriptional effects on cell cycle genes by dasatinib and nilotinib, and their EC50s in conventional cell cycle and proliferation assays. Such connectivity should be applicable across diverse cell lines: while it is always possible for drug potency to be modulated by extraneous factors, studies of these kinase inhibitors across multiple cell lines (e.g. [33],[42]) support the biochemical prediction that a single site-binder has a defined potency for its target.

Transcriptional dose responses can separate the biological effects of a multi-target compound. Whereas a microarray experiment at a single micromolar dose should identify dasatinib's impact on both DNA replication and p53 signaling, only a dose-response design revealed that the impact on the p53 pathway occurs at a micromolar dose, and thus is irrelevant to dasatinib's nanomolar anti-proliferative potency. Transcriptional dose responses also allow a more meaningful comparison to clinical parameters such as plasma concentrations observed in treated patients (200 nM for dasatinib [49]; up to 3 µM for imatinib [44]; 3.6 µM for nilotinib [50]). With regard to imatinib, our observation of numerous additional transcriptional responses as dosing increases through the micromolar range supports the assertion [43] that dosing level is critical in evaluating the relevance of *in vitro* assays or pre-clinical models to imatinib's clinical effects.

There are some limitations to the current study. First, it is impossible to measure all possible dose-responsive treatment effects in a single cell line, as not all targets are functional. In the non-ABL dependent cell line A549, we cannot evaluate and compare the on-target activity of the three clinical ABL-inhibitors. Second, the pathway analysis is limited by the quality of annotation [30]. Third, not all dose responses fit a single sigmoidal model [51]. The clustering analysis we apply for quality control occasionally reveals treatments in which groups of transcripts show sigmoidal induction at low doses but have sigmoidal down-regulation at high doses or vice versa (Text S1), presumably due to action on a second, counteractive target or process. Such behavior could be routinely identified and quantified by substituting the data model in the SDRS algorithm, permitting the subsequent analytical approaches described in this work.

In summary, we have developed new methods that enable interpretation of transcriptome behavior, including dissection of dose-dependent activities, fine differentiation between compounds, and connection with other biochemical and cellular assays. This analytical method has application at all the points of drug development where transcription profiling is currently used. In early discovery, we have compared transcriptional effects of target knockdown to the dose responses for lead compounds. As lead compounds are developed, we routinely compare the dose-dependent effects of diverse chemotypes to identify the on-target biology. Development candidates can be more clearly differentiated from a first-in-class compound, and the relative potency of an off-target activity is valuable information when assessing its importance. On transition to the clinic, transcriptional biomarkers have added validity when they are connected to target biology by a clear dose response. Investigations of approved drugs have successfully used transcriptional profiling to clarify biology (e.g. [4]); such studies can only be facilitated by a more precise linkage between dose and effects. Ultimately, dose response strategies could be combined with a compendium database of response profiles [17],[52], enabling rapid cellular categorization of new compounds.

## Methods

### Chemicals

Imatinib Cat.# PKI-IMTB-010 was purchased from Biaffin GmbH & Co KG (Cat.# PKI-IMTB-010). Nilotinib, PD-0325901 and dasatinib were provided in pure form (> = 98%) by Bristol-Myers Squibb Chemistry Division. Compounds stocks in DMSO (10 mM) were stored at −20°C and diluted in culture media before addition to cells.

### Cell viability assay

Assays were performed in triplicate. A549 cells were seeded in the interior 48 wells of 96-well plates at a density of 1×10^4^/cm^2^ in 180 µl of media, 4 hours prior to treatment with DMSO vehicle or a 11-point dose range of compound (30 µM with 3-fold dilutions down to 0.51 nM; final concentration of DMSO vehicle was 0.5% for all treatments) for 96 hours. ATP content was assayed using the CellTiter-Glo Assay (Promega, Madison, WI) with a Victor plate luminometer (Wallac, Turku, Finland).

### FACS analysis of DNA content

A549 cells were seeded in 6-well cell plates at a density of 1×10^5^/ml in 2 ml of media, 16 hours prior to treatment with DMSO vehicle or a 12-point dose range of compound (30 µM with 3-fold dilutions down to 0.17 nM; final concentration of DMSO vehicle was 0.5% for all treatments) for 23 hours. Both attached and detached cells were recovered, fixed with 0.25% ultrapure formaldehyde (Polysciences #04018) in dPBS (Ca^2+^ Mg^2+^-free; Invitrogen #14190) followed by 80% methanol. Cells were stained with dPBS/1%BSA containing propidium iodide (5 µg/ml; Sigma #P4864) and RNAse (1 µg/ml) for 30 minutes at RT in the dark. The samples were run on the FACSCanto with Diva 6.1.1software (Becton Dickinson), and data was analyzed using FlowJo 8.5.3. The experiment was performed twice (Table S7).

### Cell treatment and Affymetrix Gene Chip analysis

All handling was performed in 96-well format. Positions of the 12 levels of each treatment were randomized using an experimental design that prevented row or column effects being confounded with dose effect. A549 cells were cultured at 37°C in RPMI1640 media containing 10% heat-inactivated Fetal Bovine Serum (Mediatech, Manassas, VA). Cells were seeded at 1.7×10^5^/cm^2^ 16 hours prior to treatment with vehicle or a 12-point dose range of compound (30 µM with 3-fold dilutions down to 0.17 nM; final concentration of DMSO vehicle was 0.5% for all treatments). Cells were lysed with 1× Nucleic Acid Purification Lysis Solution (Applied Biosystems, Foster City, CA) at 4 hours or 20 hours. Total RNA was extracted using the Prism 6100 (Applied Biosystems, Foster City, CA), purified by RNAClean Kit (Agencourt Bioscience Corporation; Beverly, MA), and evaluated on a 2100 Bioanalyzer (Agilent Technologies, Santa Clara, CA). cRNA preparation and hybridization on HT-U133A 96-array plates followed manufacturer's protocols (Affymetrix, Santa Clara, CA). The CEL files were analyzed with the robust multi-array analysis (RMA) algorithm [53], obtained from www.bioconductor.org. Following quality control and removal of non-expressed probesets (Text S1), RMA values were reverse-logged (base 2) for use in sigmoidal dose response curve fitting.

### Sigmoidal dose response search (SDRS) algorithm

See also Supplementary Methods (Text S1). A standard four parameter dose response model was used to model gene expression changes in response to varying compound concentration: Y = A+(B−A)/(1+(X/C)^D^), where Y is the signal intensity value, X is compound concentration, C is the EC50, D is the slope factor, and A and B correspond to the signal intensity at low and high plateau of the curve, respectively. The approach can be viewed as a grid search, where a series of 542 values for C, distributed across the experimental dose range, are tested for every probeset on the array. For each probeset, ranges for A and B are based on signal levels in the treatment dataset. At each value of C tested, the algorithm evaluates 10,240 models against the experimental data. Goodness of fit is measured by an *F*-statistic: *F* = MSR/MSE where MSR is the mean square of the variance explained by the model and MSE is the mean square of error). For every probeset, at every C tested, the highest *F*-statistic and the corresponding A, B, D parameters are recorded. Given the normal distribution of residuals, the *F*-statistic follows an *F*-distribution, *F*(p-1, n-p), where n is the number of experimental dose points and p is the number of parameters in the model (i.e. 4: A, B, C, D). Note that the number of dose points is the most important influence on the degrees of freedom. A probeset was designated as fitted to a sigmoidal curve and corresponded to a ‘response transcript’ if its global maximal *F*-statistic (i.e. best fit) was larger than the critical *F* (95% significance level, i.e. the *F*-distribution table was consulted for 95 percentile with numerator degree of freedom of p-1 and denominator degree of freedom of n-p). For each response transcript, the values of A, B, C and D that gave rise to the maximal *F*-statistic define the optimal model and the predicted EC50. Thus the estimated EC50 presented in the SDRS report is selected from 542 possible values for C.

### Multiple test correction

After SDRS, each probeset is associated with an *F*-statistic at each of the 542 test values of C. For use in further analytical methods including data visualization, the results at a subset of 79 log-evenly distributed values for C were selected as ‘Summary Doses’ (i.e. data reduction from 542 lists to 79 lists). Each *F*-statistic was converted to the associated *P*-value. For each Summary Dose list the number of response transcripts (i.e. probesets) whose *P* value passed an FDR cutoff was calculated, using 1% increments from FDR = 1% to FDR = 35%, resulting in a 35×79 matrix. This FDR correction used the Simes procedure, which employs a series of linearly increasing critical values [54] and has been shown to control the FDR at pre-specified levels for independent test statistics [55].

### Treatment comparison and pathway analysis

All comparisons of lists were based on Fisher's exact test (FET) using the right test, which evaluates the significance of the intersection between two lists for positive association i.e. an enrichment of elements of list A in list B or *vice versa* [56]. Comparisons between two compounds were performed at each possible pair of Summary Doses (one from each compound), using the lists generated by the FDR procedure described above. (Note that there are as many as 35 distinct probeset lists for each compound at each of the 79 Summary Dose values). For each of the Summary Dose pairs, the lowest *P* value from the (maximally) 35×35 FETs was retained. The resulting 79×79 matrix is visualized as a heat map, where the depth of the color is proportional to the negative logarithm (base 10) of the *P* value.

Pathway analysis was performed using the lists generated by the FDR procedure described above. Probesets were consolidated to single gene loci to eliminate redundancy. (Note there are as many as 35 distinct gene sublists at each of the 79 Summary Doses). Each such gene list was evaluated by FET against a pathway gene list, and the lowest *P* value from the (maximally) 35 comparisons at each of the 79 Summary Dose values was retained. For comparisons that met a *P*<0.001 criterion, the resulting 79-point dataset for each pathway of interest was plotted to examine significant enrichment for pathway genes as a function of the dose range.

#### Data accession

The microarray dataset is available at ArrayExpress, E-TABM-585.

## Supporting Information

Text S1 Supplementary data and methods
(1.20 MB PDF)

Table S1 XLfit comparison
(5.98 MB XLS)

Table S2 SDRS reports for treatments
(8.27 MB ZIP)

Table S3 Loci with multiple response transcripts
(0.03 MB XLS)

Table S4 Pathway analysis
(1.51 MB ZIP)

Table S5 KEGG 04010 MAPK loci
(0.05 MB XLS)

Table S6 KEGG 04110 cell cycle response transcripts
(0.06 MB XLS)

Table S7 FACS analysis of cell cycle effects
(0.15 MB XLS)

Table S8 SDRS reports for control datasets
(8.64 MB ZIP)

Table S9 SDRS residuals for imatinib 4 hr
(5.06 MB ZIP)
