# Supplementary material for: Transcriptional Profiling of the Dose Response: A More Powerful Approach for Characterizing Drug Activities
Source: PLoS Comput Biol. 2009 Sep 18;5(9):e1000512. doi: 10.1371/journal.pcbi.1000512 (PMC2735650; doi:10.1371/journal.pcbi.1000512)

Contents:

Supplementary Data

- Evaluation of potential non-sigmoidal dose responses in raw data
- Assessment of performance: SDRS and XLfit comparison
- Effects on transcripts for genes associated with cellular metabolism
- Nilotinib specific impact on transcripts for chemokine pathways

Supplementary Methods

- Affymetrix GeneChip data quality control
- Removal of probesets with background signal
- Sigmoidal dose response searching (SDRS)

Table S10. Effects of Abl inhibitors on transcripts for genes involved in antigen processing and presentation.

Supplementary Figures

Figure S1. Comparison 4h versus 20h imatinib
Figure S2. Comparison 4h versus 20h nilotinib
Figure S3. Comparison 4h versus 20h dasatinib
Figure S4. Comparisons: all inhibitors versus each other at 4h
Figure S5. Comparisons: all abl inhibitors versus each other at 20h
Figure S6. Evaluation of signal variation in raw data to determine the types of dose response present
Figure S7. Response transcripts identified by SDRS but not XLfit are qualitatively similar to those identified by both methods.
Figure S8. Impact of dasatinib, imatinib, and nilotinib on the 'Biosynthesis of Steroids' pathway at 20 hours.
Figure S9. Impact of nilotinib on the transcripts for genes in pathways related to cytokine activity.
Figure S10. Quality control of Affymetrix data
Figure S11. Flowchart diagram representing the SDRS algorithm and its outputs in detail.

Supplementary Data

Evaluation of potential non-sigmoidal dose responses in raw data

As mentioned in the Introduction, the compounds investigated are single-site binders that are documented to produce sigmoidal dose responses in biochemical and cellular assays. However it is possible for such compounds to produce groups of transcriptional responses that would not be fitted to a single sigmoidal curve, for example due to action on multiple targets that affect the same response transcripts. An example of a non-sigmoidal dose response, observed with a different compound, is provided in **Figure S6a**. For each of the seven experimental treatments described, we examined the behavior of all probesets that showed a 2-fold range of expression values (**Fig. S6b-h**). There was no evidence of groups of transcriptional dose responses that peaked/troughed at an intermediate point of the dose range, with a gradation on either side of that dose. The examples where a single dose produces a spike in intensity for a group of probesets (without any gradation of activity in samples from neighboring doses) are very likely to be technical artifacts, given the lack of biological basis for this type of dose response. Note that we employ experimental design to ensure that handling effects (position on plate) are not confounded with dose effects. For example, the alternating patterns seen in **Figure S6b,c** are likely the result of odd-numbered doses being applied to different 96-well plates than even-numbered doses. While transcripts that exhibit more complex feedback mechanisms may exist, they are rare (i.e. they do not form a cluster of response transcripts in these heat maps) and would be difficult to distinguish from noise. In summary, investigation of sigmoidal dose responses for these compounds is appropriate.

Assessment of performance: SDRS and XLfit comparison

Grid searches and iterative approaches are both widely accepted methods for parameter optimization. To demonstrate that our application of a grid search algorithm (SDRS) can effectively identify dose responses, and provides similar model parameters to the iterative approach available to most biologists, SDRS results for imatinib treatment at 4 hours were compared to those from the commercial software XLfit. When run in batch mode, XLfit outputs a flag of 'OK!' or 'No fit' for each probeset, based on whether goodness of fit has $P < 0.05$. (XLfit can generate complete fitting statistics when one probeset is analyzed per sheet, but it is not practical to analyze microarray data in this manner). Therefore the criterion $P<0.05$ was used as a cutoff for 'response transcripts' for both methods. SDRS identified more response transcripts (2666 probesets versus 1074 for XLfit with an overlap of 1012; **Table S1**). For the 1012 shared response transcripts, the parameters of the optimal model provided in the SDRS report are typically almost identical to those generated by iterative methods (see scatter plot in **Table S1)**. Exceptions only occur where one of the curve plateaus is not present, i.e. where A or B are not well defined. In those cases, the output is dependent on the behavior of the algorithm utilized. For example, when the low plateau is missing, XLfit often generates negative estimates for A and C. Similarly, when the high plateau is missing, XLfit generates extreme values for B and C, with C often larger than the maximal experimental dose. Although there is no 'right' model in this situation, as the data is not sufficient for parameter estimation, SDRS generates more 'realistic' estimates because it imposes constraints on the parameter values based on assay data (i.e. expression values) and experimental dose range.

Regarding the response transcripts that are identified only by one algorithm: The 62 response transcripts identified only by XLfit are likely false positives, as their data had no lower

plateau and they had negative predicted EC50 values. The 1654 response transcripts identified only by SDRS include many likely true positives (i.e. response transcripts that have a high fold-change and/or well-fitted data). In all the applications described in our manuscript, SDRS response transcripts are further subselected using a multiple test correction. (Multiple test corrections are not possible with XLfit output, since unlike SDRS it does not provide a *P* value for every probeset). Therefore we also compared performance after this correction. We observed that the top 273 response transcripts from SDRS pass a 10% false discovery rate (FDR) criterion, yet 49 of the 273 were not identified by XLfit. These 49 well-fitted response transcripts identified solely by SDRS do **not** represent a distinct class of responses (for example low fold-change or occurring at the highest or lowest doses). Instead, responses of these 49 probesets are qualitatively similar to those of the 224 probesets identified by both methods (**Fig. S7**) and the majority show >1.5-fold change.

Both algorithms identify probesets that are regulated at only the highest dose of compound as response transcripts (**Fig. S7**), i.e. with no clear plateau to the response curve. SDRS assigns them an EC50 at the high end of the dose range examined, whereas XLfit assigns them an EC50 beyond the experimental doses (see scatter plot in **Table S1)**. In theory, using further high dose points would establish a plateau, but many compounds are insoluble at such levels. For any bioassay, dose ranges should be selected so that the response occurs in the middle. Where this is not possible, as in transcriptional profiling, EC50 values for responses at the extreme of the dose range are not reliable.

These results are representative of comparisons using additional datasets, and indicate that SDRS is at least as efficient as iterative regression methods for identifying a sigmoidal dose

response. Placing data-driven limits on parameter values allows SDRS to identify additional response transcripts and excludes unusable parameters such as negative EC50 values.

Assessment of robustness of SDRS to noise in dose response data

For any new method, it is also necessary to assess the robustness to naturally occurring variability in the data. We ran SDRS on ten additional 'control' datasets **(Table S8**). Five datasets were generated using permutations of 12 samples that were treated with vehicle (0.5% DMSO) for 4 hours. Each sample was randomly associated with a dose selected from the twelve actual experimental doses. Five control datasets were generated from the imatinib 4 hour dataset, by randomly permuting the dose information associated with the twelve treatments. A small percentage of probesets met a *P* value cutoff of 0.05 in every control dataset (1.6% to 7.4%, with an average of 3.1%). However, none of these probesets passed the FDR corrections used in the applications described in our manuscript, even the loosest FDR rate of 35%. We conclude that the selection of response transcript by SDRS followed by FDR correction is robust to the noise in the experimental data.

Effects on transcripts for genes associated with cellular metabolism

Each of the three ABL inhibitors affected transcripts for genes associated with cellular metabolism. This effect is most prominent in cells treated with high doses of compound for long exposures. For example, pathway analysis indicated transcript responses for a significant number of genes from the 'Biosynthesis of Steroids' pathway (KEGG:hsa00100) following 20 hours treatment by micromolar doses of all three BCR-ABL inhibitors (**Table S4** and **Fig. S8a**). At the 10 μM and 30 μM doses of compound, transcripts of genes for several enzymes of the fatty acid and cholesterol biosynthesis pathways were induced,

including ACACA, ACSL1, ACSL3, ACSL4, DHCR7, DHRS9, FADS1, FASN, FDFT1, HMGCR, IDI1, LSS, MVD, SCD, SC5DL, and SQLE. Transcripts of genes for several P450 enzymes were also affected: CYP1B1 and CYP51A1 were induced whereas CYP24A1 was repressed (**Fig. S8b**).

Nilotinib specific impact on transcripts for chemokine pathways

Pathway analysis indicated that in the micromolar dose range nilotinib produced transcript responses for a significant number of genes from the ‘Cytokine/Cytokine receptor interaction’ pathway (KEGG:hsa04060) following four hours treatment (**Table S4** and **Fig. S9a**). The 30 μM dose of nilotinib induced transcripts for CCL2, CCL5, CCL20, CXCL1, CXCL2, CXCL3, CXCL5, CX3CL1, and IL8 (**Fig. S9b**), chemokines that have been shown to be up-regulated in response to inflammation (Fischer, 2006). Nilotinib also induced several NF-κB/REL family transcription regulators, such as NF-κB1, NF-κB2, c-REL, and RELB. Two NF-κB regulators, NF-κBIA and NF-κBIE, also exhibited dose dependent response to Nilotinib treatments (**Fig. S9c**). The NF-κB/REL proteins play a major role in transcription regulation of genes responsible for both innate and adaptive immune responses including cytokine production (Li 2002, Liang 2004). Since the induction of NF-κB and related transcription regulators occurred in the same dose range as that of the cytokines, it is possible that nilotinib may induce the expression of the cytokines through the NF-κB pathway.

Pathway analysis noted that in the micromolar dose range nilotinib produced transcript responses for a significant number of genes from the ‘Antigen processing and presentation’ pathway (KEGG:hsa04612) following 20 hours treatment (**Table S4** and **Fig. S9a**). Nilotinib

regulated transcripts for several genes encoding the major histocompatibility complex, including HLA-A, HLA-C, HLA-E, HLA-F, and HLA-G. Nilotinib also affected transcripts for other genes from the antigen processing and presentation pathway, including chaperone proteins HSPA5, CALR, and CANX, peptidases CTSL1, CTSS, and LGMN, peptidase activators PSME1 and PSME2, and transporters TAP1 and TAPBP. Although our pathway analysis did not note significant number of genes from KEGG:hsa04612 among the response transcripts for dasatinib and imatinib, both inhibitors were found by visual inspection to regulate expression of some of these genes (**Table S10**). Note that identification of an impact in our pathway analysis (performed across the dose range) requires that the response transcripts have similar EC50 values.

Supplementary Methods

Affymetrix GeneChip data quality control

To determine whether cDNA synthesis had proceeded efficiently and comparably in the 111 samples used in this study, for each sample we determined the ratio of signal for a probeset at the 3' end to a probeset at the 5' end for three human transcripts (AFFX-HUMGAPDH/M33197_3_at:AFFX-HUMGAPDH/M33197_5_at (**Fig. S10a**), AFFX-HUMISGF3A/M97935_3_at:AFFX-HUMISGF3A/M97935_MB_at, and AFFX-HSAC07/X00351_3_at:AFFX-HSAC07/X00351_5_at). Ratios above the historical average are an indication of reduced efficiency in the cDNA synthesis step, giving undesirable technical variation in signal from probesets at the 5' end of transcripts. The 111 samples had normal ratios for these three parameters, indicative of satisfactory cDNA quality.

To provide quality control for the hybridization step, labeled RNA for four Escherischia coli genes (BioB at 1.5 pM, BioC at 5 pM, BioD at 25pM and CreX at 100pM) was added to the hybridization solution. There was detectable signal from AFFX-BioB probesets, and the 111 samples had equivalent signal for each control, indicative of satisfactory hybridization.

In order to detect samples that were outliers (single samples that account for a high degree of the variation in the data) Principal Components Analysis (PCA) was performed for each experimental group using data for all 22218 probesets. In all cases, the first three principal components account for ≤ 30% of the variation (**Fig. S10b,c,d**), and no individual sample accounted for the majority of this variation. At 20 hours, dose-dependent effects on variation were evident, most prominently among the Dasatinib samples.

Removal of probesets with background signal

Removal of probesets with background signal intensity has a strong impact on the false discovery rate. With data from the Affymetrix MAS 5 algorithm, up to 50% of the probesets on the HG133A array are typically scored as 'absent' and can be removed. Using data from the RMA algorithm, we performed a more conservative trim. The probesets removed were different for each treatment (for example, the data for dasatinib at 4 hours was separated from the data for dasatinib at 20 hours). For each treatment, the probeset list was sorted based on the maximal expression value for the probeset across all twelve samples. The lowest 15th percentile of probesets was removed. For the remaining 18883 probesets, a mean signal value was obtained from the vehicle-treated control samples. Thus for each probeset in each of the seven treatment datasets there were 13 data points (1 mean value from the vehicle-treated controls and 12 from treatments with compound).

Sigmoidal dose response searching (SDRS)

A flowchart diagram that details each step in the SDRS algorithm and its outputs is provided in **Figure S11**. For every probeset, the range for the parameter A was determined based on the six lowest expression values of the probeset. The average and standard deviation of these six values were computed and the range of A was set to be mean $\pm$ 2 x standard deviation. The search step for A was set to be ¼ of the standard deviation. Similarly, the range for the parameter B was determined using the six highest expression values and the step was also ¼ of its standard deviation. The parameter D was set to be between -6.3 and 6.3, with a step of 0.3. (In reality, D can vary from $-\infty$ to $+\infty$. However, when the absolute value of D is > 6,

additional increments have only marginal impact on the estimates of the other three parameters.)

The scan started at the lowest compound dose, 0.17 nM, moved up the dose scale with a multiple of 1.18 from the previous scan dose, and ended when it reached the highest dose of 30,000 nM. The scan step was set to a maximum of 60 nM. This allowed a more precise estimate of the EC50 value. There were 542 search points in total for each dataset using this method.

Goodness of fit was measured by an F statistic: F = MSR/MSE where MSR is the mean square of the variance explained by the model and MSE is the mean square of error.

Source of variation	Sum of Squares	Degrees of freedom	Mean Square
Regression	$SSR = \Sigma(y_{computed} - y_{average})^2$	p - 1	$\frac{SSR}{(p-1)}$
Error	$SSE = \Sigma(y_{observed} - y_{computed})^2$	n - p	$\frac{SSE}{(n-p)}$
Total	$SST = \Sigma(y_{observed} - y_{average})^2$	n - 1	

The residuals for SDRS applied to data from a 4 hour treatment with imatinib are presented in **Table S9**. While it is difficult to assess normality from only 12 values, and it is impossible to examine all 18,833 distributions, distribution of residuals seems unbiased. Given normality of the residuals, the *F*-statistic follows an *F*-distribution.

The 95% confidence interval of the EC50 can be found in a two step process. First, a line corresponding to the *F*-critical value for α=0.05 is drawn, which typically intersects at two points with the *F*-statistic plot (see **Figure 1A**). Second, the two intersect points are projected onto the dose axis, which defines the lower and upper boundaries of the confidence interval.

References

Fischer, M. et al. Mast cell CD30 ligand is upregulated in cutaneous inflammation and mediates degranulation-independent chemokine secretion. J. Clin. Invest. 116, 2748-56 (2006).

Li, Q., Verma, I.M. NF-kappaB regulation in the immune system. Nat. Rev. Immunol. 2, 725–34 (2002).

Liang, Y., Zhou, Y., and Shen, P. NF-kappaB and its regulation on the immune system. Cell. Mol. Immunol. 1, 343-50 (2004).

Table S10. Effects of Abl inhibitors on transcripts for genes involved in antigen processing and presentation. Transcripts annotated to the 'Antigen processing and presentation' pathway (KEGG:hsa04612) that by visual inspection exhibit sigmoidal dose response to dasatinib, imatinib, and nilotinib at 20 hours. A value of '-1' indicates repression by a compound, whereas '1' indicates induction.

Gene	Description	Dasatinib	Imatinib	Nilotinib
CALR	Calreticulin			1
CANX	Calnexin	1		1
CTSL1	Cathepsin L1	1	1	1
CTSS	Cathepsin S			1
HLA-A	Major histocompatibility complex, class I, A	1		1
HLA-C	Major histocompatibility complex, class I, C	1		1
HLA-DMA	Major histocompatibility complex, class II, DM alpha	1		
HLA-DME	Major histocompatibility complex, class II, DM beta		-1	
HLA-E	Major histocompatibility complex, class I, E	1		1
HLA-F	Major histocompatibility complex, class I, F	1		1
HLA-G	HLA-G histocompatibility antigen, class I, G	1		1
HSP90AA1	Heat shock protein 90kDa alpha (Cytosolic), class A member 1	-1	1	
HSPA1A	Heat shock 70kDa protein 1A	-1	1	
HSPA1B	Heat shock 70kDa protein 1B		1	
HSPA5	heat shock 70kDa protein 5 (glucose-regulated protein, 78kDa)			1
LGMN	Legumain	1		-1
NFYB	Nuclear transcription factor Y, beta		1	
NFYC	Nuclear transcription factor Y, gamma	1		
PSME1	Proteasome (prosome, macropain) activator subunit 1 (PA28 alpha)			1
PSME2	Proteasome (prosome, macropain) activator subunit 2 (PA28 beta)			1
TAP1	Transporter 1, ATP-binding cassette, sub-family B (MDR/TAP)	1	1	1
TAPBP	TAP binding protein (tapasin)			1

Figure S1. Characterization of the overall transcriptional response to kinase inhibitor imatinib, and a comparison between the four hour and twenty hour timepoints.

(a-b) The number of probesets whose F statistic for goodness of fit to a sigmoidal curve passes the given FDR criterion. In each heatmap, the horizontal axis contains the SDRS Summary Doses, and the vertical axis contains one row for each of the 35 FDR criteria applied (panel **(b)** is rotated 90 degrees to clarify its relationship to panel **(c)**). The results shown are from SDRS applied to data from a four hour treatment **(a)** or a 20 hour treatment **(b)** with imatinib. **(c)** The negative logarithm of *P* values from Fisher's exact tests comparing response transcripts for imatinib at the two timepoints. Values displayed are the lowest *P* value observed in right tests performed between the (maximally) 35 lists of probesets that underlie each value of C in panels **(a)** and **(b)**. The horizontal axis contains the SDRS Summary Doses generated using data for imatinib treatment for 4 hours, and the vertical axis contains the SDRS summary doses generated using data for imatinib treatment for 20 hours.

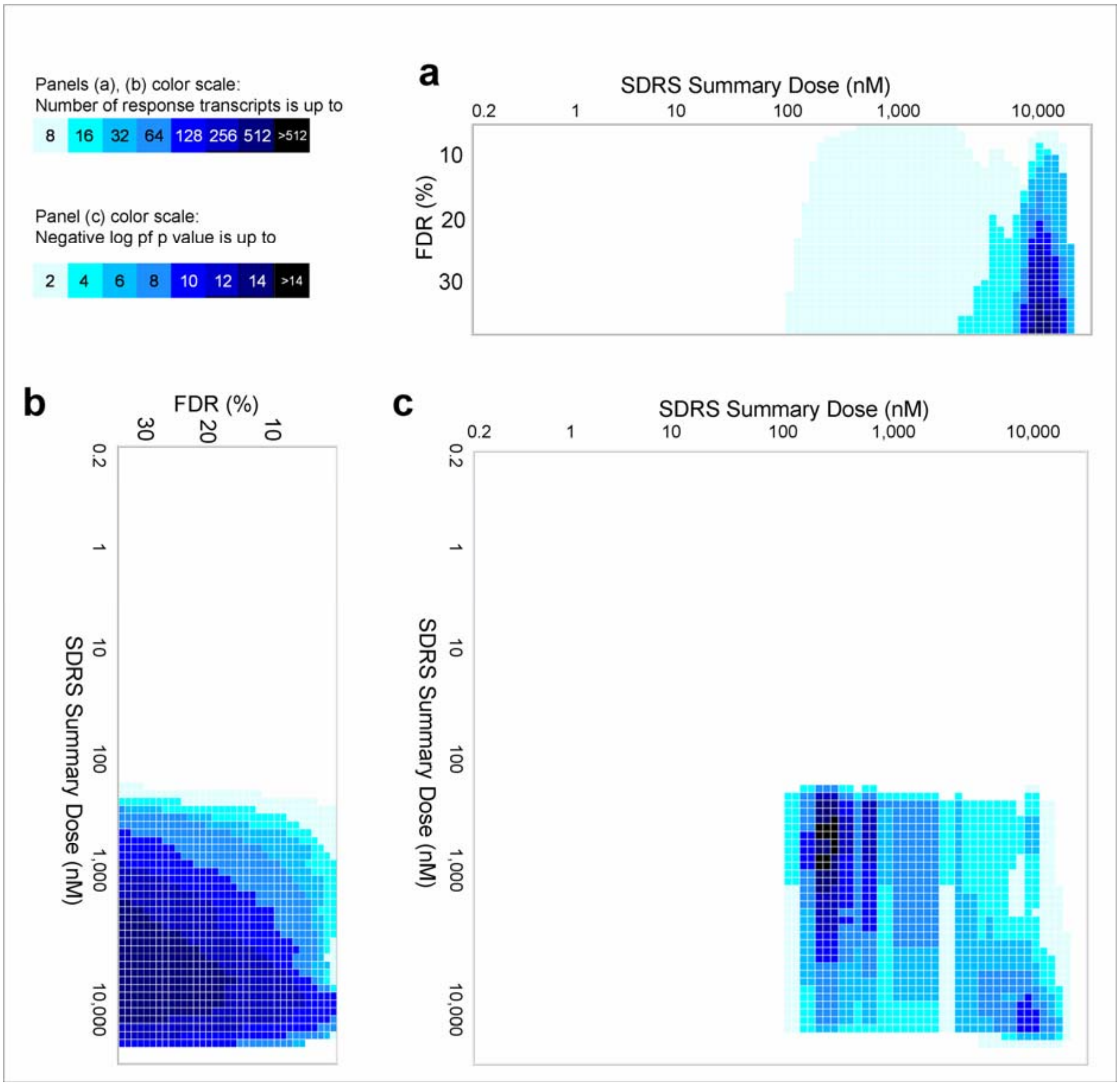

Figure S2. Characterization of the overall transcriptional response to kinase inhibitor nilotinib, and a comparison between the four hour and twenty hour timepoints.

(a-b) The number of probesets whose F statistic for goodness of fit to a sigmoidal curve passes the given FDR criterion. In each heatmap, the horizontal axis contains the SDRS Summary Doses, and the vertical axis contains one row for each of the 35 FDR criteria applied (panel **(b)** is rotated 90 degrees to clarify its relationship to panel **(c)**). The results shown are from SDRS applied to data from a four hour treatment **(a)** or a 20 hour treatment **(b)** with nilotinib. **(c)** The negative logarithm of *P* values from Fisher's exact tests comparing response transcripts for nilotinib at the two timepoints. Values displayed are the lowest *P* value observed in right tests performed between the (maximally) 35 lists of probesets that underlie each value of C in panels **(a)** and **(b)**. The horizontal axis contains the SDRS Summary Doses generated using data for nilotinib treatment for 4 hours, and the vertical axis contains the SDRS Summary Doses generated using data for nilotinib treatment for 20 hours.

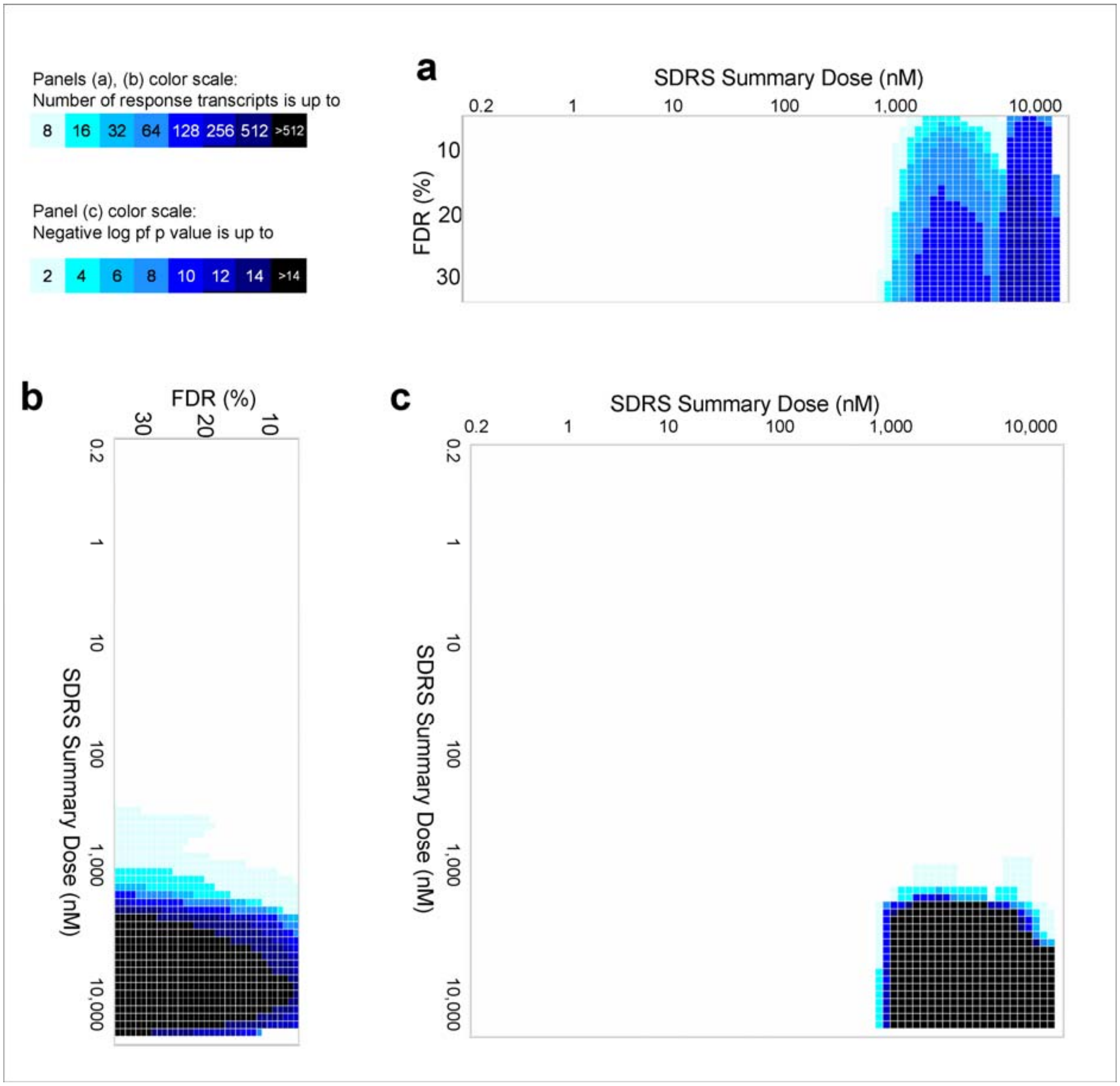

Figure S3. Characterization of the overall transcriptional response to kinase inhibitor dasatinib, and a comparison between the four hour and twenty hour timepoints.

(a-b) The number of probesets whose F statistic for goodness of fit to a sigmoidal curve passes the given FDR criterion. In each heatmap, the horizontal axis contains the SDRS Summary Doses, and the vertical axis contains one row for each of the 35 FDR criteria applied (panel **(b)** is rotated 90 degrees to clarify its relationship to panel **(c)**). The results shown are from SDRS applied to data from a four hour treatment **(a)** or a 20 hour treatment **(b)** with dasatinib. **(c)** The negative logarithm of *P* values from Fisher's exact tests comparing response transcripts for dasatinib at the two timepoints. Values displayed are the lowest *P* value observed in right tests performed between the (maximally) 35 lists of probesets that underlie each value of C in panels **(a)** and **(b)**. The horizontal axis contains the SDRS Summary Doses for dasatinib treatment for 4 hours, and the vertical axis contains the SDRS Summary Doses for dasatinib treatment for 20 hours.

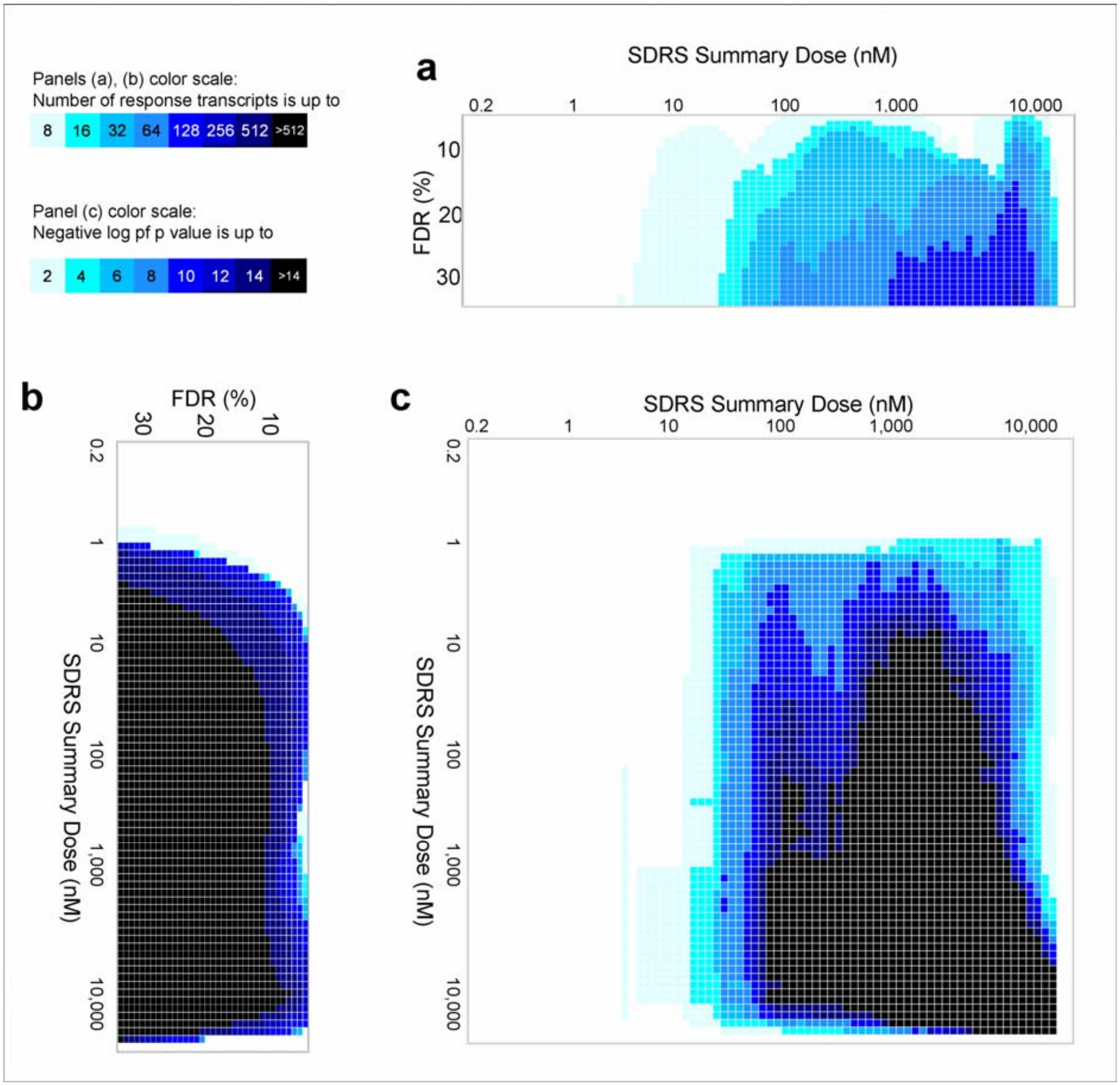

Figure S4. Comparison between transcriptional responses to PD0325901, imatinib, nilotinib and dasatinib at the four hour timepoint. For reference, the distribution of the transcriptional response is shown in **(a)** and to the right of **(b)**: these are smaller images of the data presented in **Figure 2b** and **Supplementary Figures 1-3**. **(a)** The number of probesets whose F statistic for goodness of fit to a sigmoidal curve passes the given FDR criterion. In each heatmap, the horizontal axis contains the SDRS Summary Doses, and the vertical axis contains one row for each of the 35 FDR criteria applied. **(b)** The negative logarithm of *P* values from Fisher's exact tests comparing response transcripts for the compounds indicated. Values displayed are the lowest *P* value observed in right tests performed between the (maximally) 35 lists of probesets that underlie each value of EC50 in panel **(a)**.

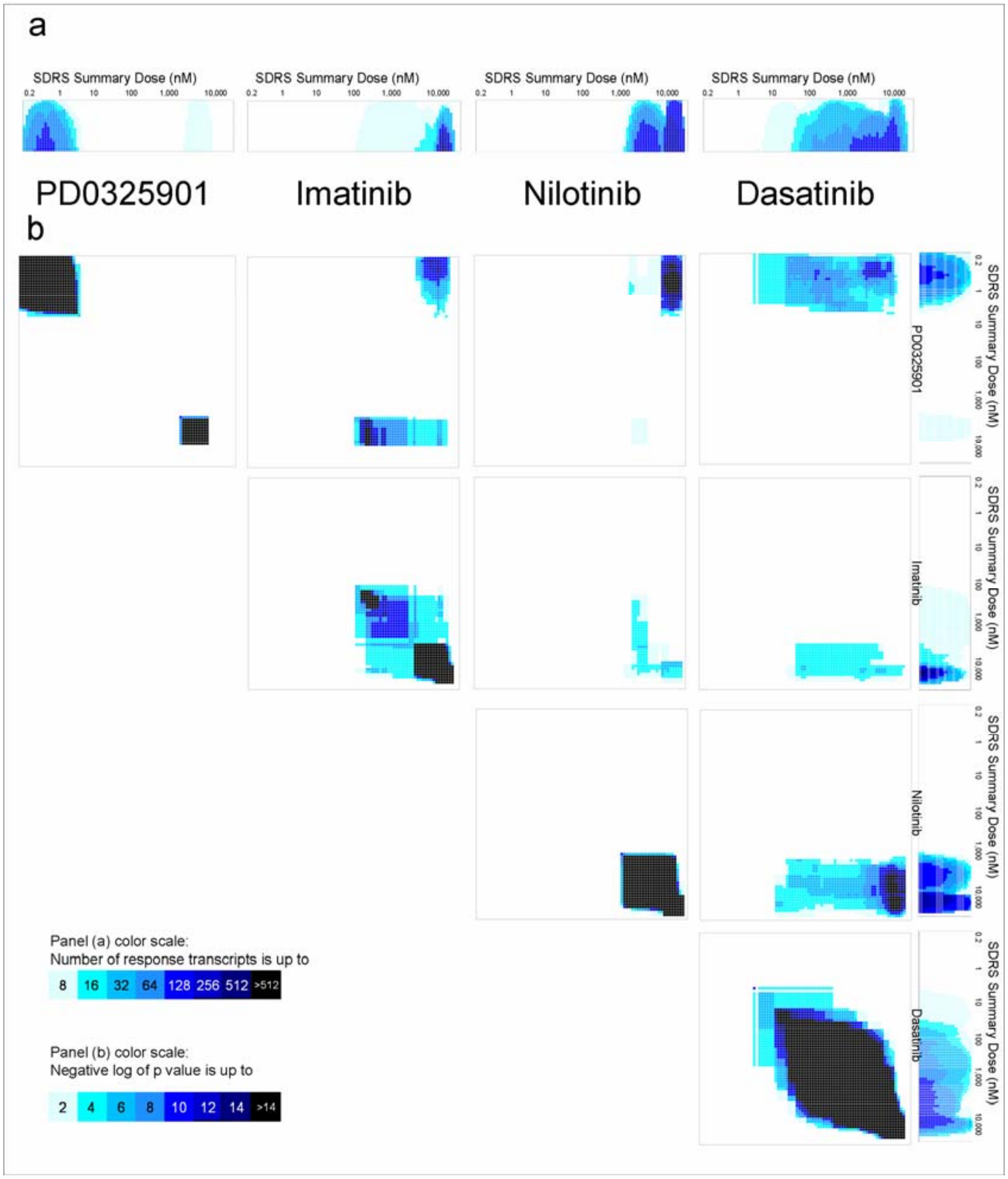

Figure S5. Comparison between transcriptional responses to imatinib, nilotinib and dasatinib at the 20 hour timepoint. For reference, the distribution of the transcriptional response is shown in **(a)** and to the right of **(b)**: these are smaller images of the data presented in **Figures S1-S3**.

(a) The number of probesets whose F statistic for goodness of fit to a sigmoidal curve passes the given FDR criterion. In each heatmap, the horizontal axis contains the SDRS Summary Doses, and the vertical axis contains one row for each of the 35 FDR criteria applied. **(b)** The negative logarithm of *P* values from Fisher's exact tests comparing response transcripts for the compounds indicated. Values displayed are the lowest *P* value observed in right tests performed between the (maximally) 35 lists of probesets that underlie each value of EC50 in panel (a).

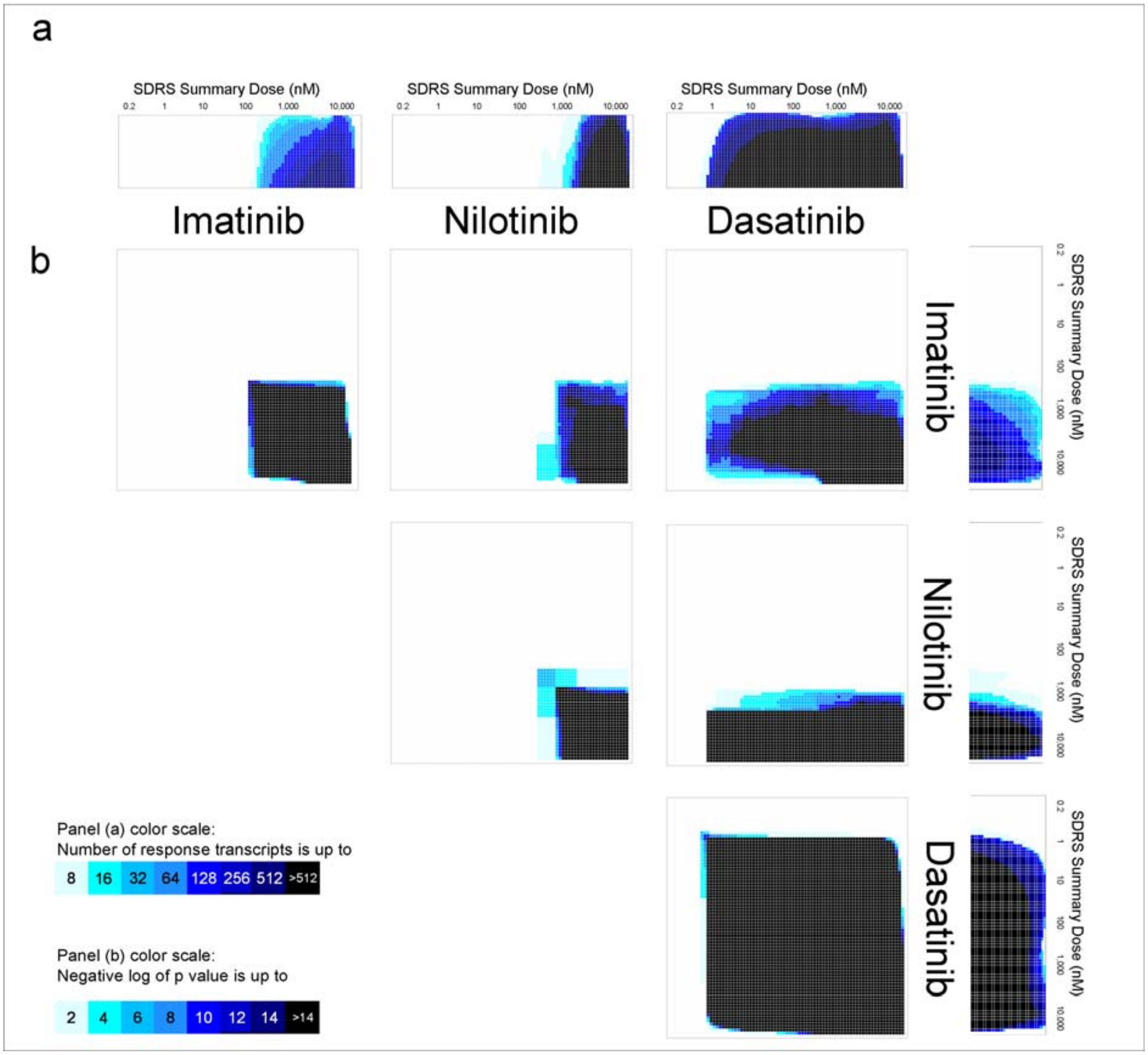

Figure S6. Evaluation of signal variation in raw data to determine the types of dose response present

The heat maps show signal intensity for the probesets (in rows) across the twelve doses of compound (columns). The probesets were identified as having a 2-fold range of signal intensity in the RMA data for a given treatment. a) example of non-sigmoidal dose responses b) PD0329501 4h, 260 probesets c) imatinib 4h, 129 probesets d) imatinib 20h, 379 probesets e) nilotinib 4h, 224 probesets f) nilotinib 20h, 670 probesets G) Dasatinib 4h, 178 probesets h) Dasatinib 20h, 1051 probesets. For visualization, RMA signal intensity data was reverse-logged and scaled from zero (blue) to one (red) for each probeset, and rows were clustered based on Euclidean distance by agglomerative average linkage.

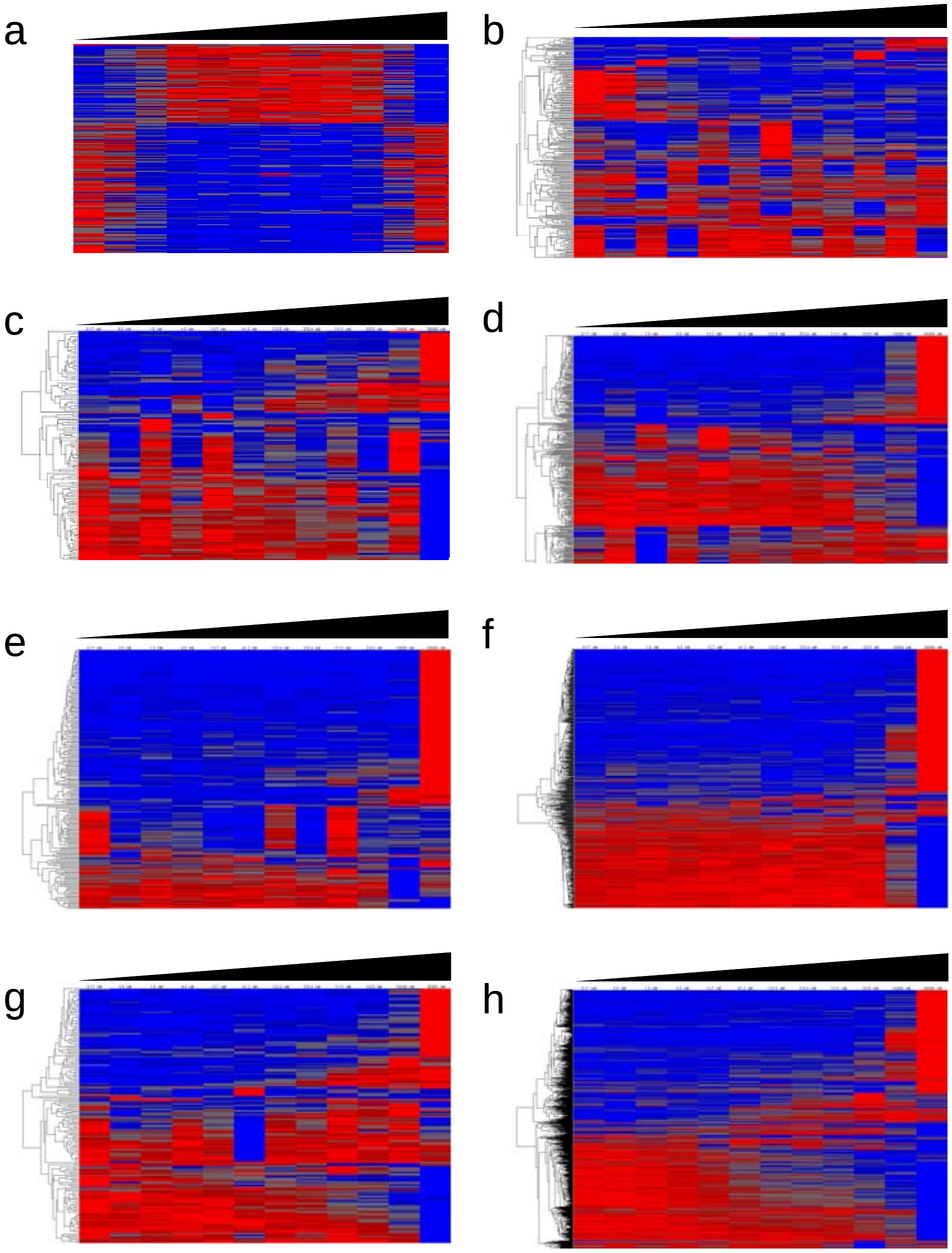

Figure S7. Response transcripts identified by SDRS but not XLfit are qualitatively similar to those identified by both methods.

The heat map shows signal intensity for 273 probesets (in rows) across twelve doses of imatinib (columns). The 273 probesets were identified by SDRS in data from treatment with imatinib for four hours, and pass a 10% FDR criterion for fitting to a sigmoidal dose response curve. Top panel contains 49 probesets that were **not** fitted to a sigmoidal dose response curve by XLfit (P <0.05); bottom panel contains 224 probesets that were fitted by both algorithms. For visualization, RMA signal intensity data was reverse-logged and scaled from zero (blue) to one (red) for each probeset, and rows were clustered based on Euclidean distance by agglomerative average linkage.

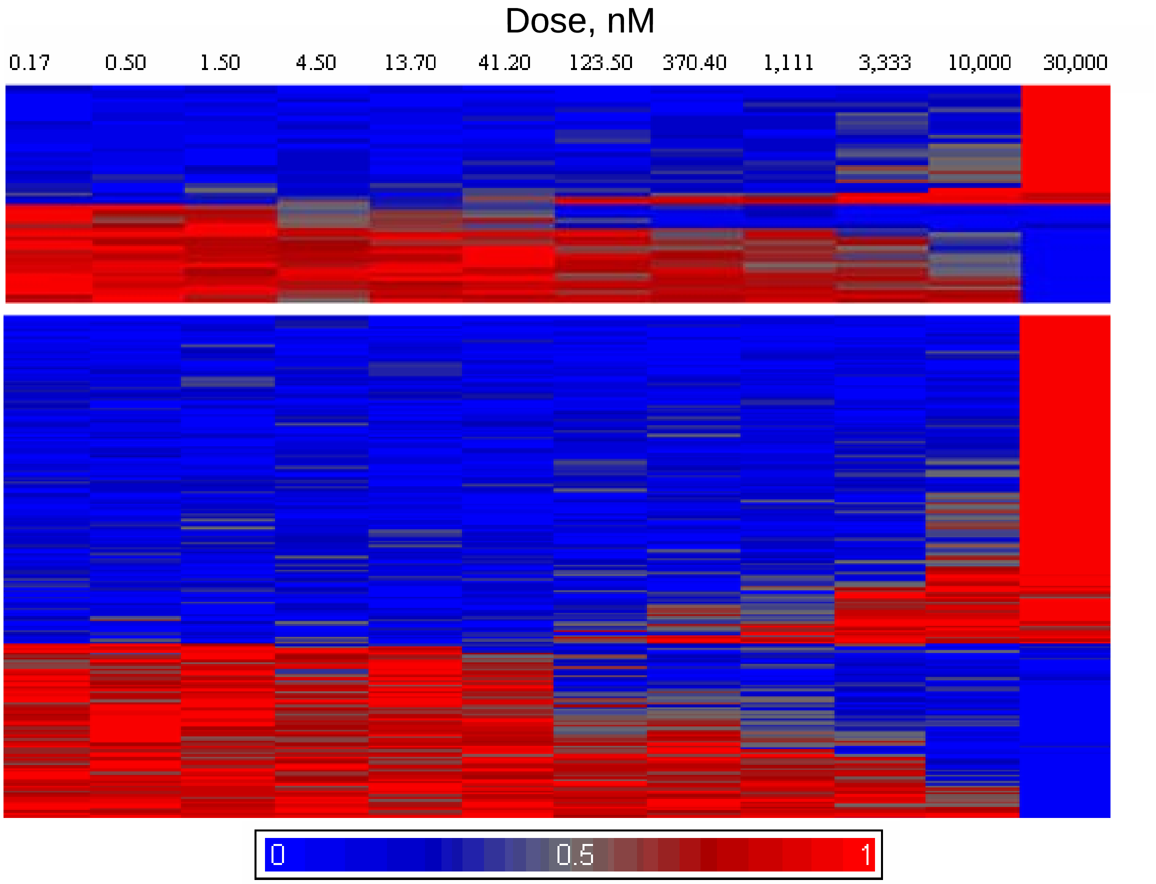

Figure S8. Impact of dasatinib, imatinib, and nilotinib on transcripts for genes in the 'Biosynthesis of Steroids' pathway and P450 enzymes at 20 hours.

(a) Plot of significance values obtained from Fisher's exact tests performed between the lists of gene loci corresponding to response transcripts (1 to 35% FDR), and a list of gene loci representing the 'Biosynthesis of Steroids' pathway (KEGG:hsa00100). The *x* axis represents the SDRS Summary Doses. The *y* axis shows the negative logarithm of the lowest *P* value obtained. **(b)** Experimental data for probesets corresponding to ACACA, ACSL1, DHCR7, DHRS9, FADS1, FASN, LSS, MVD, SCD, SQLE, annotated to KEGG:hsa00100, and P450 enzymes (CYP1B1, CYP24A1, CYP51A1), with dose response curves obtained from the optimal model parameters established by SDRS.

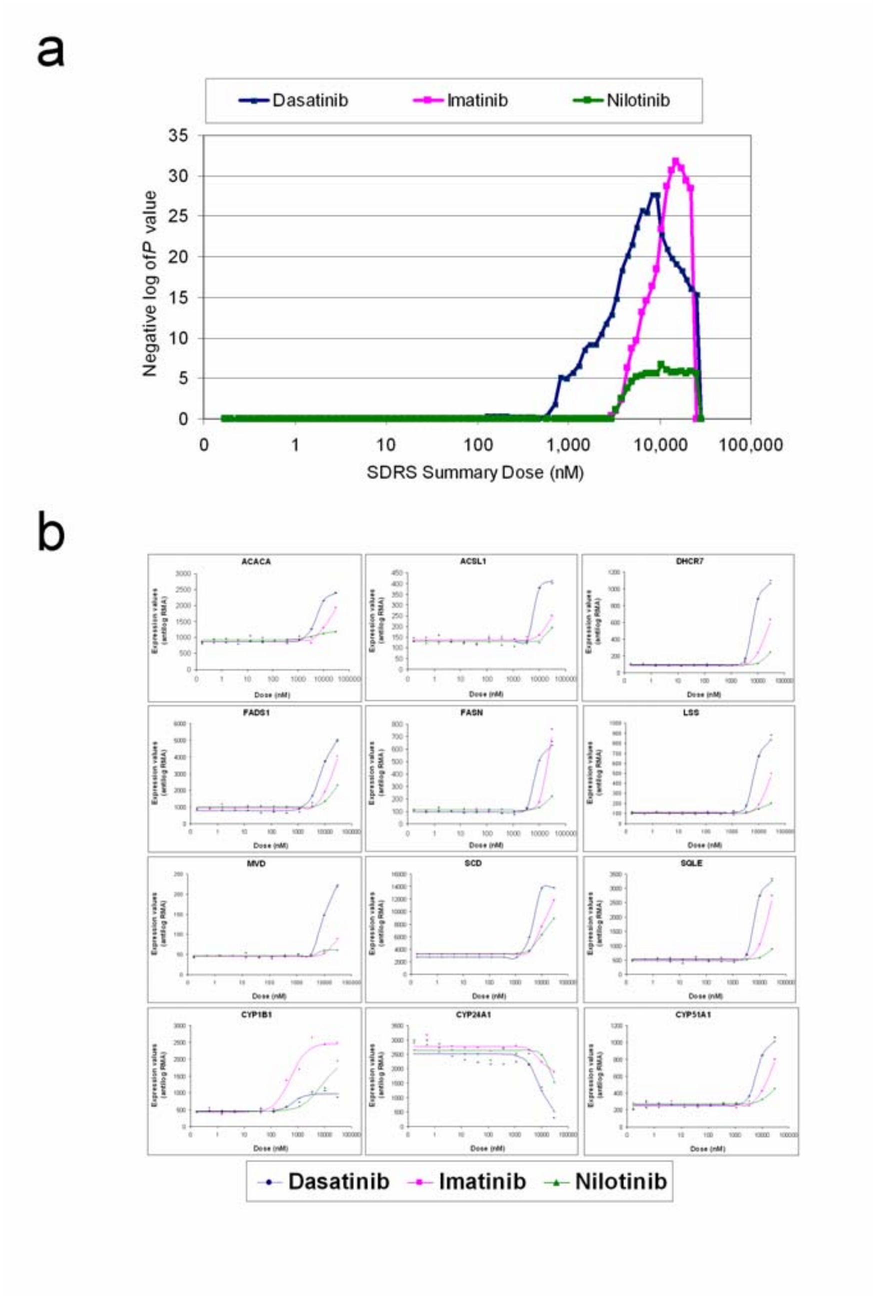

Figure S9. Impact of nilotinib on the transcripts for genes in pathways related to cytokine activity. (a) Plot of significance values obtained from Fisher's exact tests performed between the lists of gene loci corresponding to response transcripts (1 to 35% FDR), and list of gene loci representing the KEGG and GO pathways indicated. The x axis represents the SDRS Summary Doses. The y axis shows the negative logarithm of the lowest *P* value obtained. (b) Experimental data for probesets corresponding to CCL2, CCL5, CCL20, CXCL1, CXCL2, CXCL3, CXCL5, CX3CL1, and IL8 with dose-response curves obtained from the optimal model parameters established by SDRS. (c) Experimental data for probesets corresponding to NF-κB1 and NF-κB2, and to their regulators NF-κBIA and NF-κBIE, with dose-response curves obtained from the optimal model parameters established by SDRS.

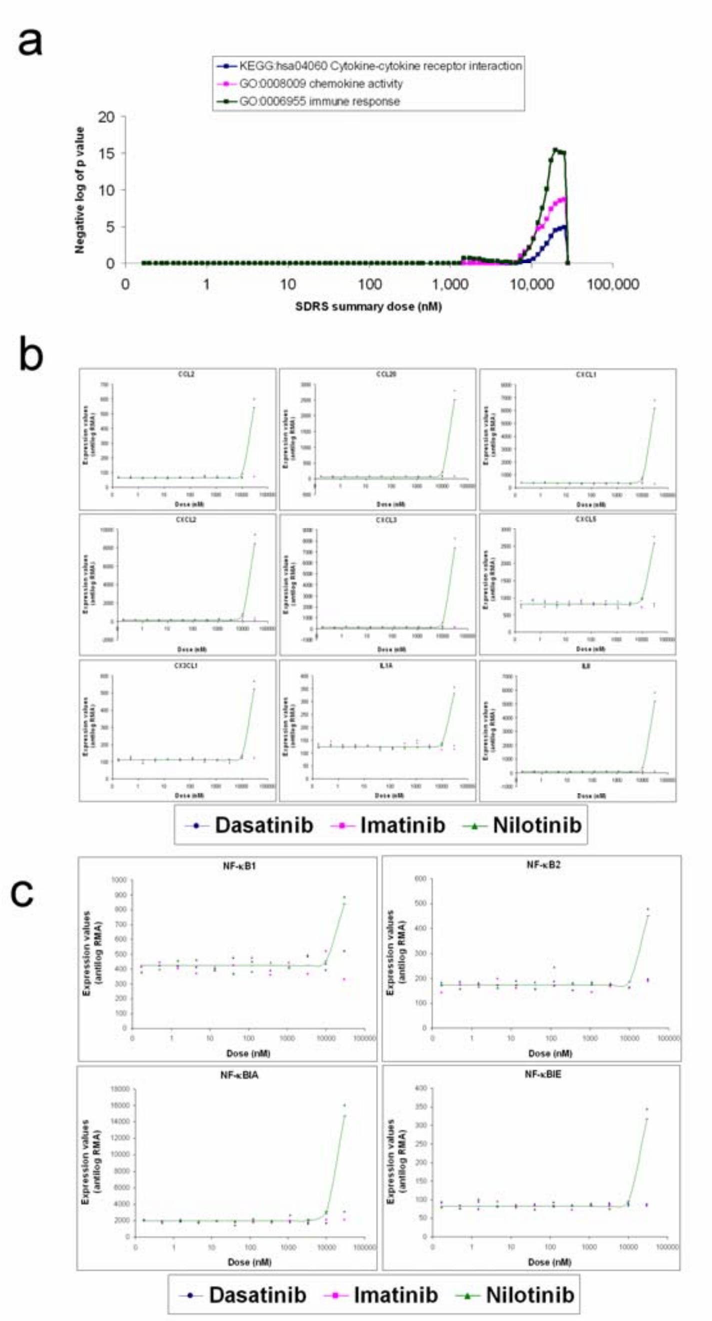

Figure S10. Quality control of Affymetrix data
(a) Ratio of signal for probeset at the 3' end of the human GAPDH transcript (AFFX-HUMGAPDH/M33197_3_at) to signal for probeset at the 5' end of the GAPDH transcript (AFFX-HUMGAPDH/M33197_5_at) for all samples. Single letter codes D, I, N, P denote dasatinib, imatinib, nilotinib and PD0325901, respectively. Blue slopes indicate dose scales. **(b-d)** Principal Component Analysis (PCA) using data for all 22,218 probesets. **(b)** Data from A549 cells treated with dasatinib, imatinib or nilotinib for four hours at the levels indicated. Seven samples treated with 0.5% DMSO (vehicle controls) are also shown. **(c)** Data from A549 cells treated with dasatinib, imatinib or nilotinib for 20 hours at the levels indicated. Nine samples treated with 0.5% DMSO (vehicle controls) are also shown. **(d)** Data from A549 cells treated with PD0325901 for four hours at the levels indicated. Six samples treated with 0.5% DMSO (vehicle controls) are also shown.

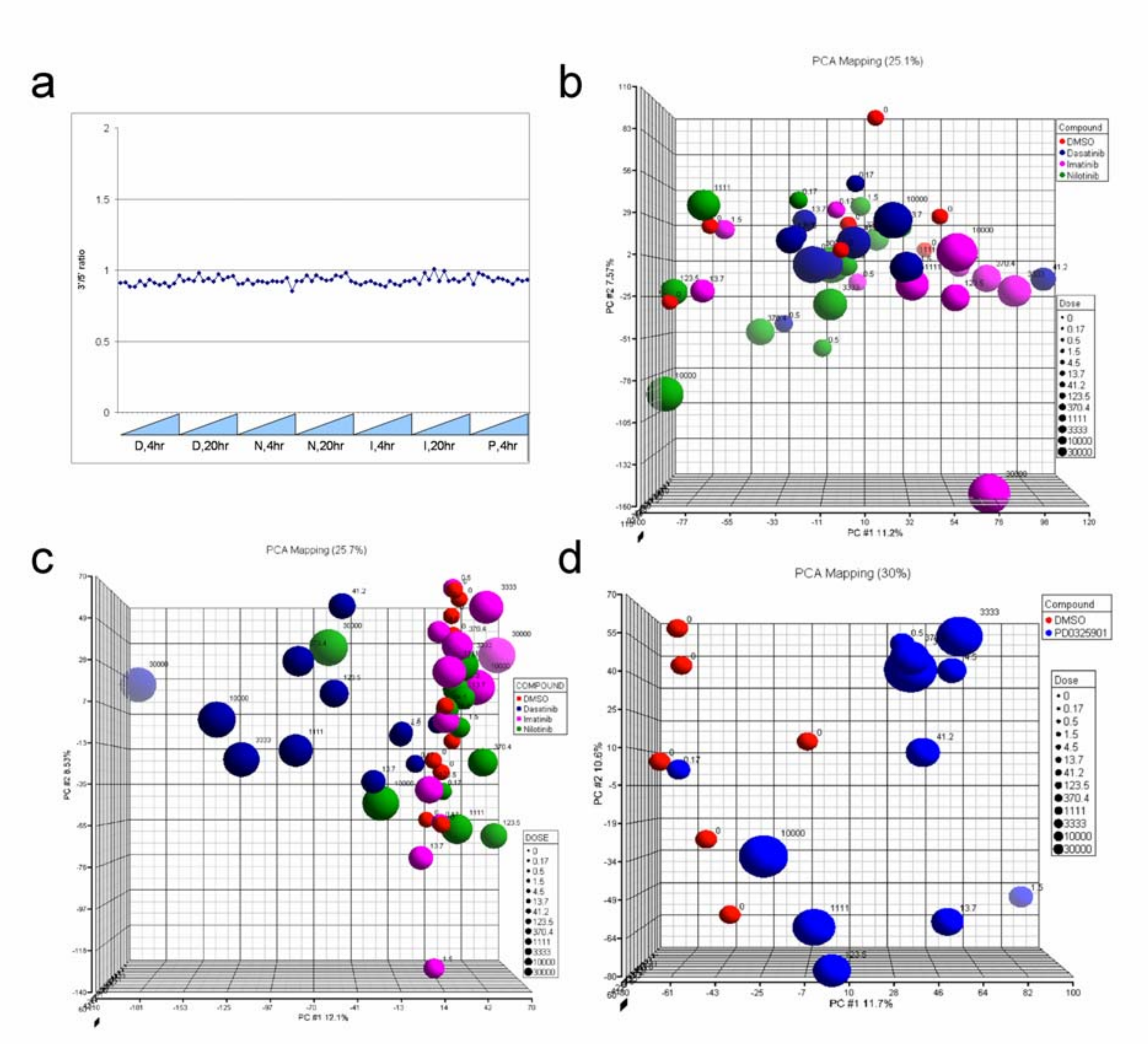

Figure S11. Flowchart diagram representing the SDRS algorithm and its outputs in detail.

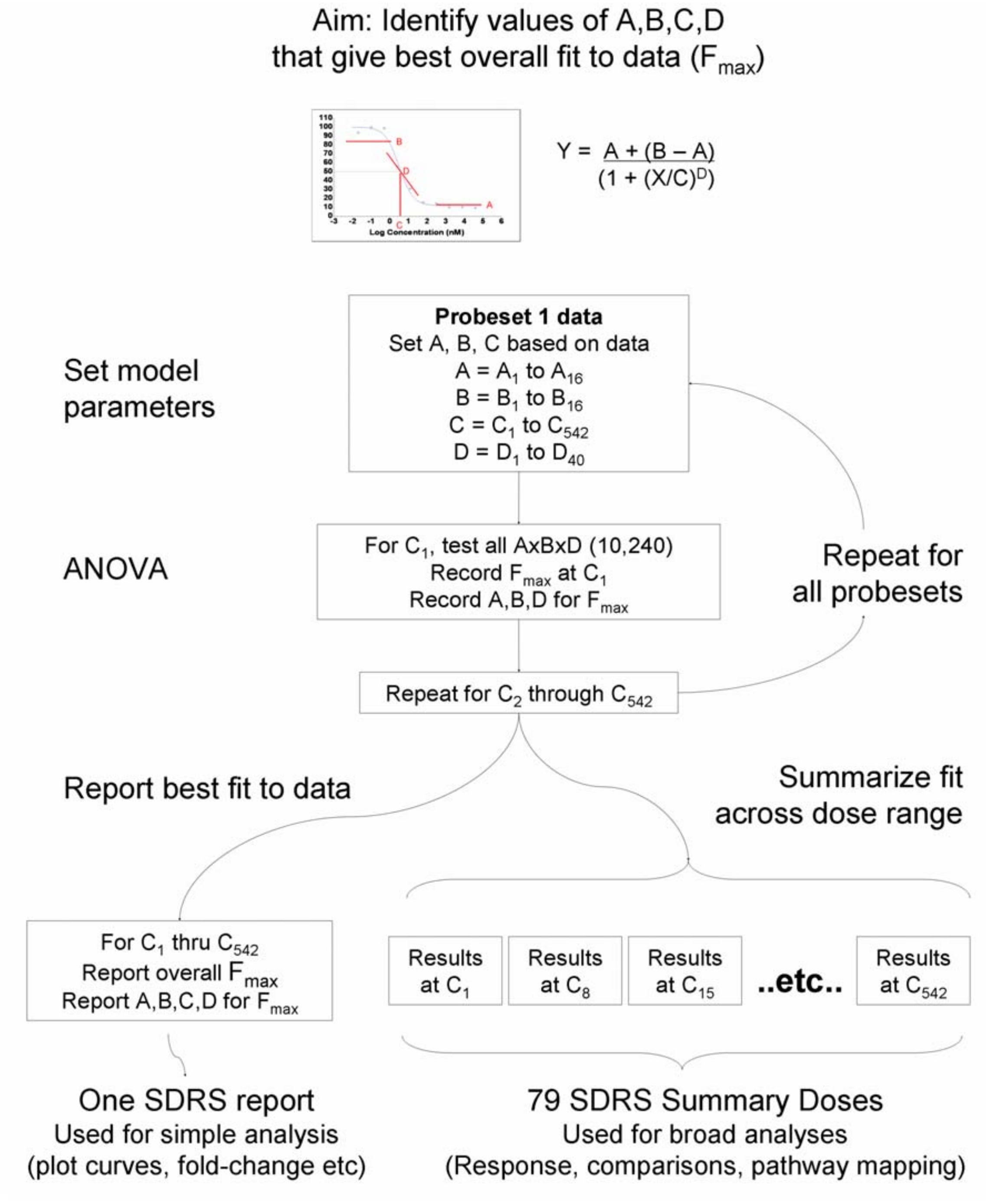

Supplement: Text S1 — Supplementary data and methods (1.20 MB PDF) [file pcbi.1000512.s001.pdf]
